# A Silicon‐Stereogenic Silanol ‐ ^18^O‐Isotope Labeling and Stereogenic Probe Reveals Hidden Stereospecific Water Exchange Reaction

**DOI:** 10.1002/chem.202202935

**Published:** 2022-11-10

**Authors:** Felix Langenohl, Jonas Rösler, Sebastian Zühlke, Jan‐Lukas Kirchhoff, Carsten Strohmann

**Affiliations:** ^1^ Inorganic Chemistry TU Dortmund University Otto-Hahn-Straße 6/6a 44227 Dortmund Germany; ^2^ Center for Mass Spectrometry (CMS) TU Dortmund University Otto-Hahn-Straße 6/6a 44227 Dortmund Germany

**Keywords:** chirality, configuration, isotopic labelling, silanols, silicon

## Abstract

A silicon‐stereogenic aminosilanol was isolated in excellent diastereomeric ratio and the absolute configuration was determined. The silanol is configurative and condensation stable in solution and shows stereoselective transformations with a clean stereospecific pathway in follow‐up reactions, which leads to the isolation of a silicon‐stereogenic zinc complex and siloxane compounds. Investigations with ^18^O‐labelled water and mass spectrometry analysis revealed an otherwise hidden exchange of oxygen atoms of silanol and water in solution that proceeds with retention of the configuration at the silicon center. This novel combination of a stereochemical probe and isotopic labeling in a silicon‐stereogenic compound opens new analytic possibilities to study stereochemical courses of reactions with the aid of chiral silanols mechanistically.

## Graphical abstract

**Silanols are important** industrial building blocks for the functionalization of surfaces. In this research, one silicon‐stereogenic silanol was selectively synthesized and analyzed. By using an innovative combination of this stereochemical probe and isotope labeling, a configurationally stable chiral silanol was studied, providing new insights into mechanistic studies.

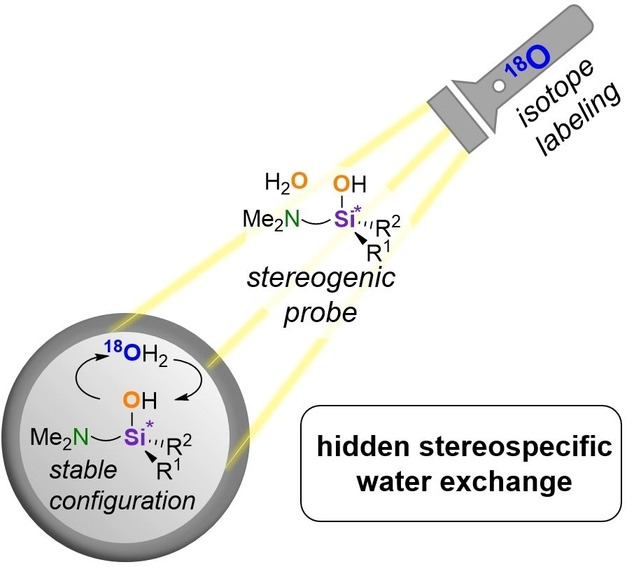

## Introduction

Silanols are a central class of compounds in silicon chemistry and are widely used in synthesis,[^1^, ^2^, ^3^, ^4^, ^5^] catalysis[^6^, ^7^, ^8^] and as bioactive agents.[^9^, ^10^, ^11^, ^12^] They also play a key role as intermediates in the development of Si−O based materials, such as silicones and siloxanes.^[13]^ For the development of more complex and efficient materials, chiral components are an important tool to ensure better control of the processes, taking nature‘s synthesis performance as a role model. In addition, especially Si‐chiral silanols could serve as an efficient chiral probe, providing new insights into downstream processes such as silanol condensation, which would be useful for silicone and siloxane material chemistry to optimize and synthesize improved products.^[14]^ In the past, the concept of using chiral silanes as mechanistic probes has been demonstrated successful regarding other systems.[^15^, ^16^, ^17^]

However, the preparation of Si‐chiral compounds has proven challenging in the past due to limited access to suitable prochiral or chiral precursors.[^18^, ^19^, ^20^, ^21^, ^22^, ^23^, ^24^, ^25^] Combined with the metastability of silanols, which can lead to undesirable self‐condensation processes to siloxanes, this creates a major synthetic hurdle. Therefore, the synthetic access is challenging.[^26^, ^27^, ^28^, ^29^, ^30^, ^31^, ^32^, ^33^, ^34^, ^35^, ^36^, ^37^, ^38^, ^39^, ^40^, ^41^, ^42^] Although the documented synthetic strategies provide access to chiral silanols, many works focus on the synthetic aspect, while studies on the stability and subsequent chemistry of these components are still underrepresented. These are important for silanols, since the configuration of silanols in solution is not necessarily stable, as described for a bioactive silanol, documenting a racemization of the Si‐chiral compounds in aqueous medium. This leads to the possibility of a decrease in the biological activity and thus the usability of this component.^[27]^ In parallel, water represents a central role in silanol chemistry, as it is formed in all condensation processes.

Despite these initial findings on the limited configurational stability of silanols in solution, this aspect was not investigated further. Questions arise here, whose answers are essential in order to maximize the potential applications of chiral silanols. How stable is the configuration of chiral silanols in solution? Which subsequent reactions can be realized without loss of stereo information to make further functional components accessible?

**Scheme 1:**
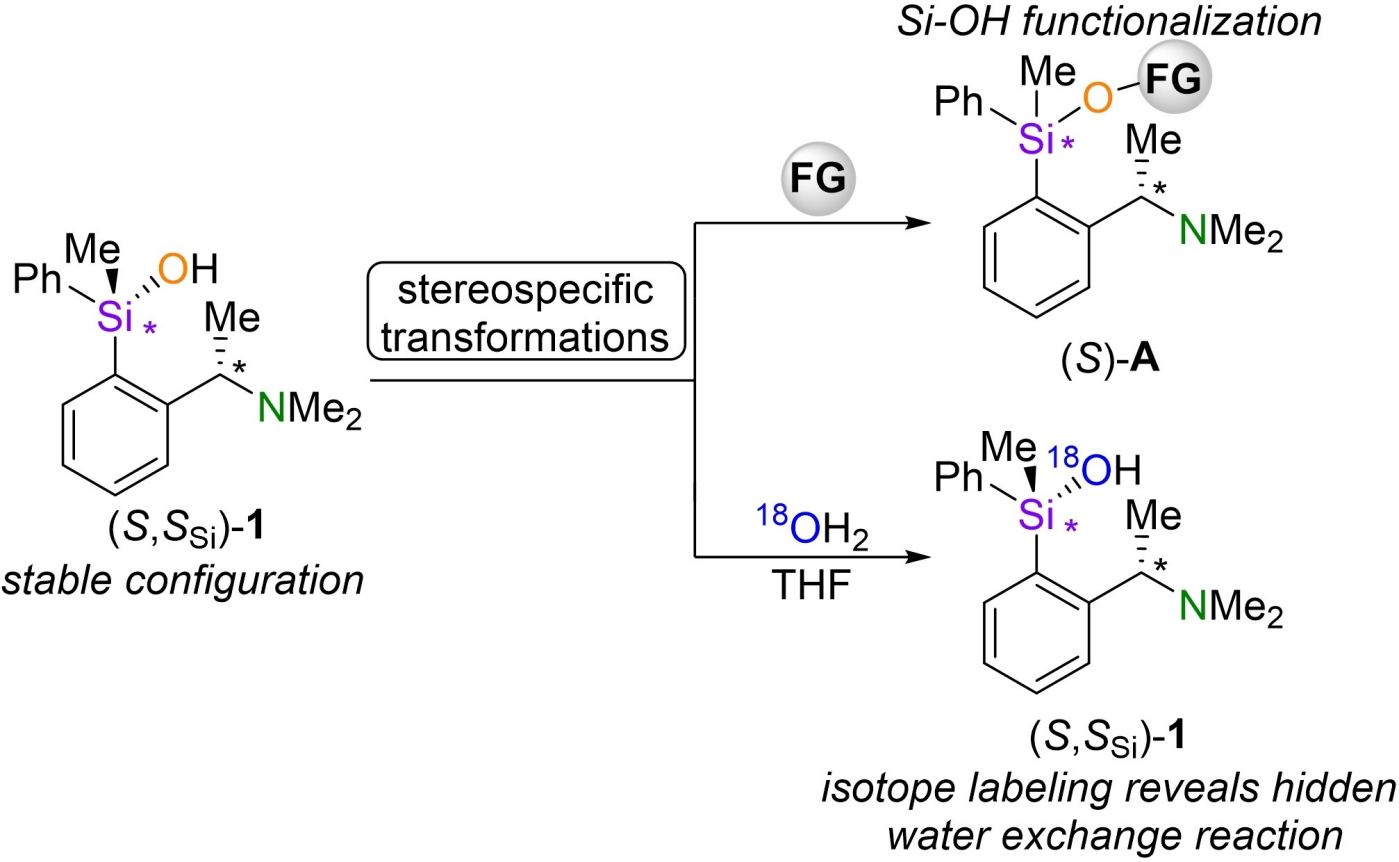
Easy access to silanol **1** in high diastereomeric purities with stable configuration in solution enables stereospecific transformation reactions. Isotope labelling experiments show exchange reactions of silanol **1** with water without loss of stereo information; functional group (FG).

In this article we want to address some aspects of these questions by presenting the synthesis, isolation, and stability studies of Si‐chiral silanol **1** (Scheme 1) in high diastereomeric purity.

Thus, it can be used as an efficient mechanistic probe whose potential can be extended by ^18^O‐isotopic labelling experiments to investigate otherwise invisible exchange processes with water. NMR experiments show that silanol **1** is configurational stable in benzene, THF and Et_2_O and does not show condensation to the corresponding siloxane. In addition, the Si‐OH‐function can be stereospecific transformed into corresponding functional groups (FG) like siloxides and siloxanes.

## Results and Discussion

**Scheme 2:**
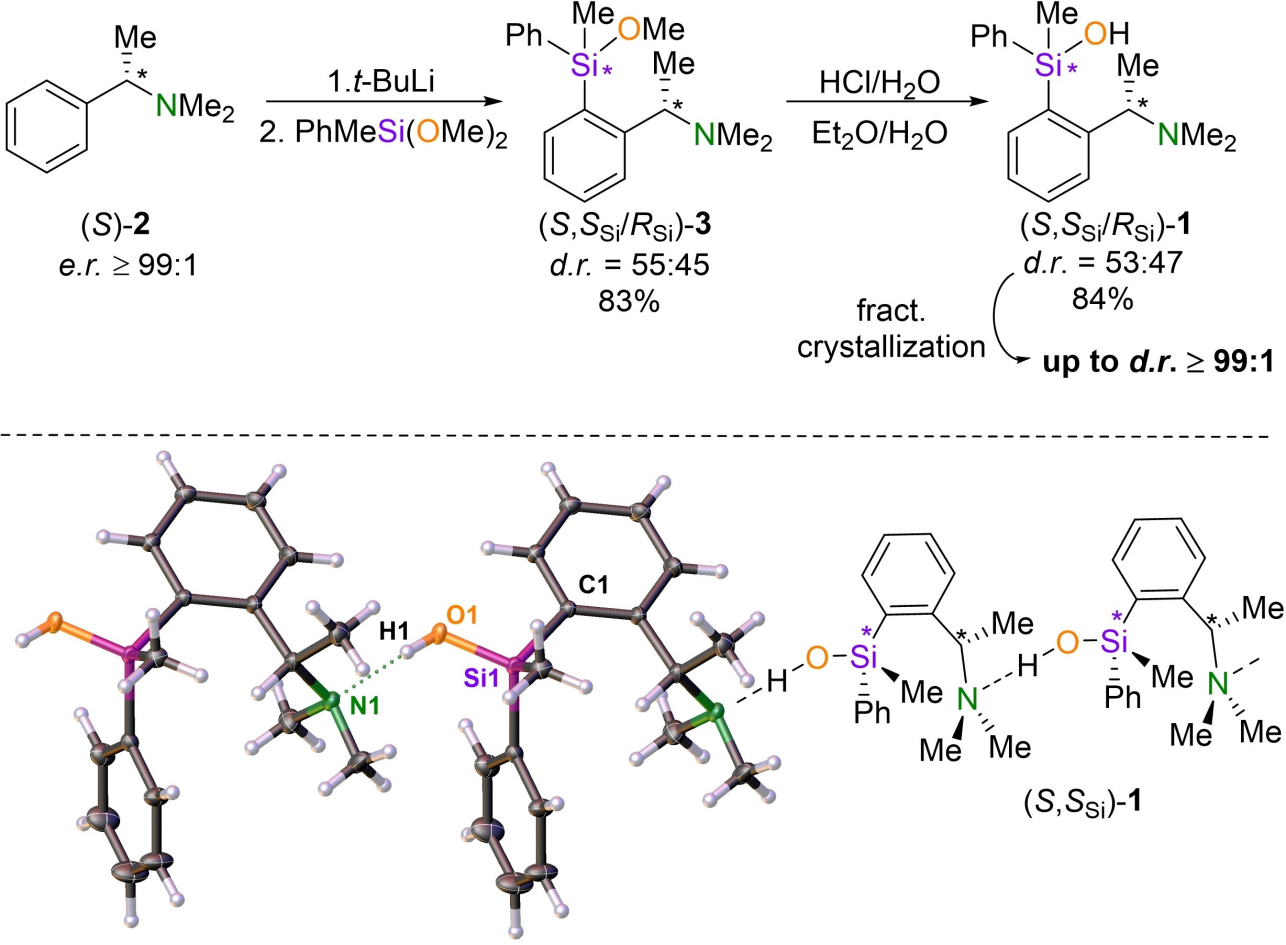
Top: Synthesis of silanol **1**. (83 % isolated yield methoxysilane **2**; 84 % isolated yield silanol **1** as diastereomeric mixture, 37 % isolated yield silanol **1** with *d.r*.≥99 : 1 related to the amount of used methoxysilane) Bottom: Molecular structure of silanol **1**.^[44]^ Thermal ellipsoid representation with 50 % probability level. Selected bond lengths [Å] and ‐angels [deg]: Si1−C1 1.8844(8), Si1−O1: 1.6323(7), O1−H1: 0.81(3), H1−N1 1.91(3), O1−N1 2.7170(10), O1−H1−N1 172(2), Si1−O1−H1 119.3(17); Symmetry code: −1+X, Y, Z.

Silanol **1** was synthesized by the acid‐catalyzed hydrolysis of the corresponding methoxysilane (Scheme 2). The methoxysilane was prepared via ortho‐lithiation from the commercially available enantiomerically pure amine **2**, analogous to the synthesis methodology known from the literature.^[43]^

After hydrolysis, silanol **1** was initially obtained as a diastereomeric mixture. Subsequent crystallization of silanol **1** revealed a drastic enrichment of one diastereomer in a kinetic resolution, allowing easy isolation of this isomer with diastereomeric ratios of up to *d.r*.≥99 : 1. By using single crystal X‐ray structural analysis the absolute configuration of the enriched species was identified as (*S*,*S*_Si_). Silanol **1** crystallizes in the space group *P*2_1_. The quality of the crystal structure allowed the localization and free refinement of all protons. Two silanol molecules were observed in the asymmetric unit. These pack with the molecules generated by symmetry operations along the a‐axis to form long, chain‐like structures and are connected by intermolecular hydrogen bonds between the hydroxyl group of silanol **1** and the amine function of the next silanol.

**Figure 1:**
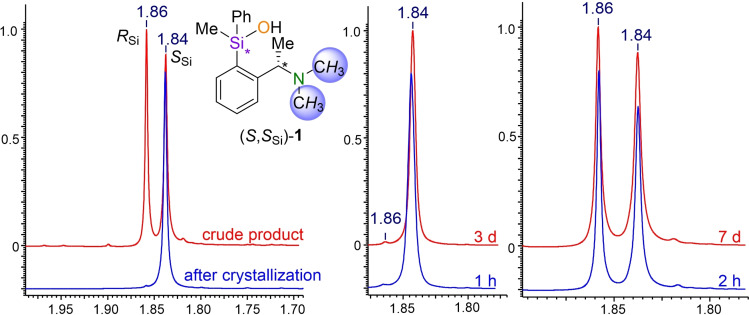
^1^H NMR spectra of silanol **1** in C_6_D_6_ (y‐axis: normalized intensity, x‐axis: chemical shift in ppm). Left: diastereomeric mixture of crude product and diastereomeric pure compound after crystallization. Center: Confirmation of configurative stability for the diastereomeric pure compound. Right: Confirmation of configurational stability for the diastereomeric mixture. Timescale related to the dissolving of silanol **1** in C_6_D_6_.

In the following, conditions were sought in which silanol **1** would be configurationally stable. Moreover, silanol **1** does not show condensation to the siloxane to provide the conditions for clean conversion reactions. For this purpose, the silanol was dissolved in different solvents and ^1^H‐ and ^29^Si NMR spectra of the compound were recorded over a period of several days (Figure 1). A detailed measurement of silanol **1** in C_6_D_6_ showed no change in NMR spectra and thus complete stability against condensation reaction and configurational changes over a period of one week. ^29^Si NMR experiments in non‐deuterated Et_2_O (4 days) and THF (8 days) also showed high stability of silanol **1** in the time periods studied.

After the configurational stability of silanol **1** under the conditions was confirmed, it was converted in first transformation reactions. The focus was on the selective synthesis of chiral siloxanes and siloxides. In this case, access to Si‐chiral representatives is of great interest, especially for the development of new materials. In addition, the metallated siloxides often show outstanding catalytic activity and access to Si‐stereogenic ligands could open the development of new components for chiral catalysis.[^45^, ^46^, ^47^]

**Scheme 3:**
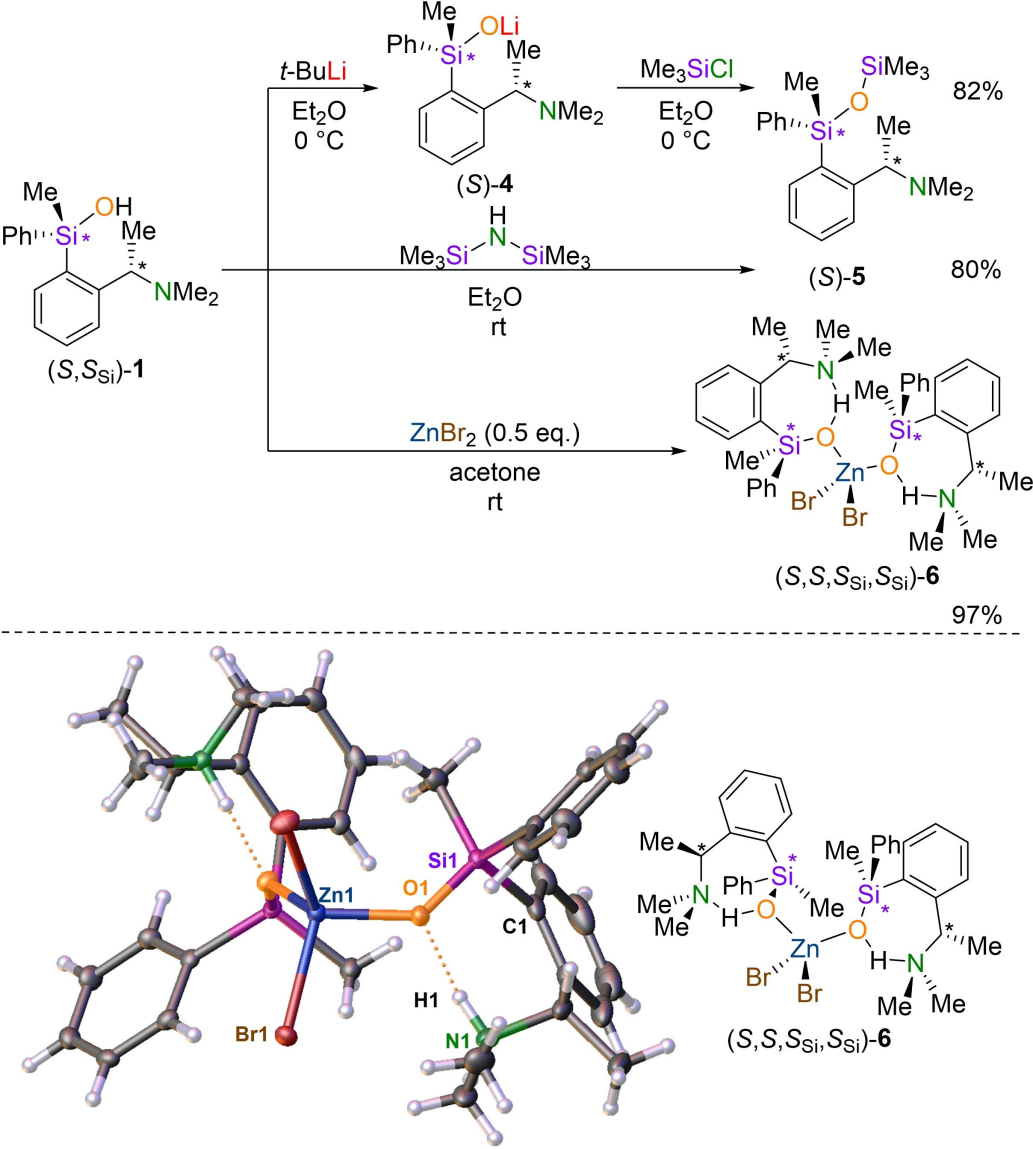
Stereospecific transformations of silanol **1**. Synthesis via lithium‐siloxide **4** to siloxane **5** (yield 82 %); direct synthesis with hmds to siloxane **5** (isolated yield 80 %); synthesis of zinc complex **6** with zinc bromide (isolated yield 97 %). Below: Molecular structure of complex **6**.^[48]^ Thermal ellipsoid representation with 50 % probability level. Hydrogen atoms integrated in hydrogen bonds were localized and freely refined on the electron density map. Selected bond lengths [Å] and ‐angels [deg]: Si1−O1 1.6112(14), Zn1−O1 1.9439(14), Zn1−O2 1.9578(13), Zn1−Br1 2.4043(4), O1−N1 2.627(2), O1−H1 1.70 (3), N1−H1 0.93(3), N1−H1−O1 169(3), O1−Zn1−O2 113.11(6). Si1−O1−Zn1 133.15(9). (*S*)‐**4** and (*S*)‐**5** were obtained using a stereospecific process with *S*_Si_:*R*_Si_ *d.r*.≥99 : 1. Expected retention on silicon is shown in the synthetic scheme to underline the stereospecificity, despite the absolute configurations these two compounds are unknown.

In the first experiment, *t*‐butyllithium was added to silanol **1** to form the corresponding lithium siloxide and was directly converted with Me_3_SiCl to obtain siloxane **5**. Here, using NMR spectroscopy, a clean reaction to the siloxane was observed over both reaction steps without changes of diastereomeric ratio of (*S*)‐**5**. This provides access to stereochemically pure siloxanes (82 %). An alternative synthesis strategy was carried out in which silanol **1** was reacted directly with hexamethyldisilazane to give the corresponding siloxane **5**. And again, the product was obtained in high yield (80 %) using a stereospecific process (Scheme 3).

The reaction of silanol **1** with zinc bromide led to the formation of the zinc complex **6** which was isolated in high yields in crystalline form. Here, the single crystal X‐ray structure analysis showed the formation of a dimeric complex on which the configurations at the silicon centers are analogous to silanol **1** (*S*_Si_)‐configured. Thus, the absolute configuration of zinc complex **6** was determined as (*S*,*S*,*S*_Si_,*S*_Si_).

The complex **6** crystallizes in the space group *P*2_1_2_1_2_1_ together with acetone. In the asymmetric unit two zinc complexes and two solvent molecules were observed. The protons are involved in hydrogen bonds, analogous to solid state structure of silanol **1**. The structure is thus similar to known solid‐state structures of other zinc complexes of this type.[^49^, ^50^]

For a deeper investigation of configurational stability, following the configurational instability of a silanol in an aqueous media described by tacke,^[27]^ the configurational stability of silanol **1** in the presence of water should be investigated in more detail.

**Scheme 4:**
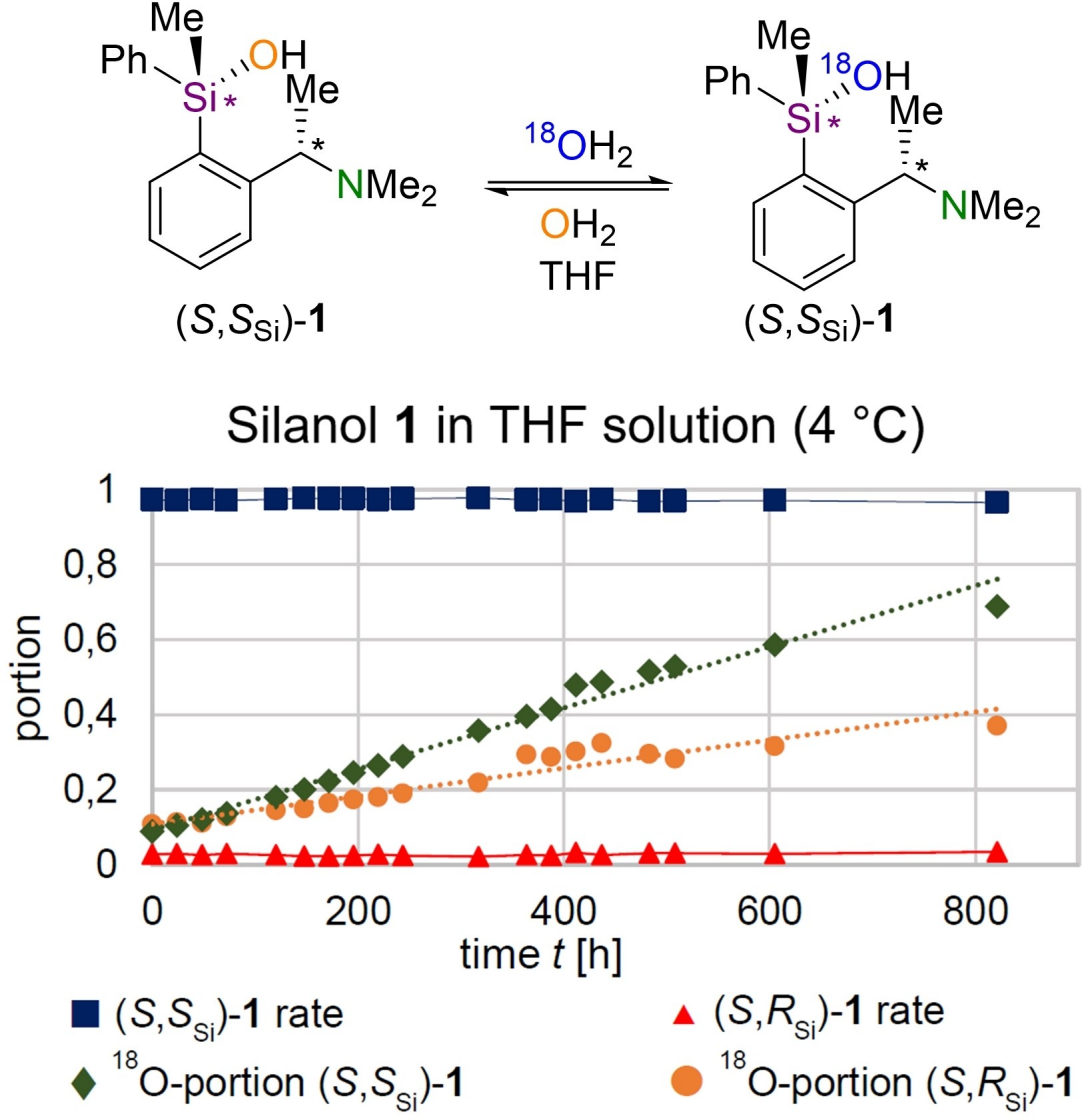
Exchange reactions of silanol **1** in THF (4 °C). The diastereomeric ratio of (*S*_Si_/*R*_Si_) remains constant under the experimental conditions; The proportions of ^18^O in both isomers of silanol **1** increase sharply during the experimental period. Conditions: *c*_[silanol]_=10 mg/mL, *c*[^18^OH_2_]=30 μL/mL. An analogue room temperature experiment shows corresponding results in shorter time.

For this purpose, a THF solution of silanol **1** was mixed with isotopically labeled H_2_^18^O (97 %). Additional to the mechanistic information obtained from the ratio of the diastereomers (*S*_Si_/*R*_Si_), further insights can be gained by an isotopic labeling of the silanol, which can reveal the exchange processes between silanol **1** and water (Scheme 4). The combination of these two mechanistic “probes” therefore results in an extremely powerful analytical tool that can pro‐vide deep insights into ongoing reactions. For this experiment, a suitable MS method had to be developed, since silanol **1** condenses with the silica phase during GC measurements. In parallel, the separation of the diastereomers and the determination of the components had to be ensured. In this case, a detectable species was obtained by derivatizing the silanol to siloxane **5**. For the method used, a calibration of the substances was carried out and measuring points were determined in triplicate in order to generate valid results. Silanol **1** was used for the experiments only in stereochemically enriched form [*d.r*. (*S*_Si_/*R*_Si_)=98 : 2] to be able to detect the minor diastereomer as well. To ensure constant experimental conditions, the solution was stored in the refrigerator (T=4 °C) for the duration of the experiment.

During the experiment, samples were taken at regular intervals and the corresponding ratios of the diastereomers (*S*_Si_/*R*_Si_) were determined after derivatization to siloxane **5**. In parallel, the most intense oxygenated molecule fragments were used to determine the respective proportions of ^18^O in the corresponding diastereomers by GC/EI‐MS. The evaluation of the experiments shows an unexpectedly long stability of the configuration at the silicon center, which at first glance suggests no exchange with water. However, at the same time, the incorporation of large amounts of ^18^O into the diastereomers of silanol **1** is observed, making the labeled species the major components in solution at the end of the experimental period. Since this exchange occurs without loss of stereochemical information, it can be concluded that the reaction proceeds cleanly with retention of the configuration.

These show that a stable configuration is not necessarily associated with frozen reactivity, since initial experiments of chiral silanol **1** in THF with ^18^O‐labeled water show a steady exchange of the hydroxyl groups of silanol **1**, which proceeds with retention of the configuration at the silicon center and thus ensures stability of the configuration over a long period of time. In the two diastereomers, the ^18^O‐fraction increases at different velocities and the first results obtained in this study indicate that the (*S*_Si_)‐isomer exchanges with water faster than the (*R*_Si_)‐isomer of **1**. It is possible that the amino substituent influences the exchange process through the formation of hydrogen bonds.

## Conclusion

In summary, we were able to demonstrate access to a Si‐chiral silanol that is stable to configurational changes and to condensation reactions in solution, which can be transformed in clean stereochemical transformations to siloxanes and siloxides. In addition, an isotope labeling experiment revealed hidden exchange processes of water with silanol **1** that proceed in THF with retention of the configuration, thus ensuring long term stability of the configuration in the aqueous environment. The combination of a stereochemical probe and isotopic labeling experiments opens the possibility for large‐scale mechanistic studies of the central processes of silanols, which could provide access to new synthesis opportunities for silanol‐based materials.

## Experimental Section

**General remarks**: All reactions with oxygen‐ and moisture‐sensitive compounds were performed under an atmosphere of argon with Schlenk‐techniques in dried solvents. The solvents were purified and dried by distillation over sodium and storage under argon atmosphere. Commercially available reagents were used without further purification except for phenylmethyldimethoxysilane, which was distilled and stored over molsieve (4 Å) in argon atmosphere. The NMR spectra were measured on a Bruker Avance III HD NanoBay – 400 MHz, 500 MHz Bruker Avance NEO, 600 MHz Bruker Avance III HD and 500 MHz Agilent Technologies DD2 spectrometer at 25 °C. Chemical shifts (*δ* in ppm) are referred to tetramethylsilane (TMS), with the deuterium signal of the solvent serving as internal lock and the residual solvent signal as additional reference ^1^H NMR *δ* (C_6_D_5_H)=7.16 ppm). Signals were assigned with the help of HSQC experiments. For the multiplicities following abbreviations were used: s=singlet, br=broad signal, d=doublet, q=quartet. In diastereomeric associations, diastereomeric signals were assigned to the major diastereomer (D_maj_) and the minor diastereomer (D_min_) if distinguishable. For spectra measured in non‐deutered solvents, a capillary with C_6_D_6_ was added and served as an internal standard. ^13^C NMR Spectra were measured with broadband decoupling and referred to the signal of the solvent [{^1^H}^13^C NMR *δ* (C_6_D_6_)=128.39 ppm]. {^1^H}^29^Si NMR spectra were as inverted gated with TMS as external standard. Single crystal X‐ray diffraction for compounds **1** and **6** were conducted on a *Bruker D8 Venture* four‐circle diffractometer by *Bruker AXS GmbH* using a PHOTON II CPAD detector by *Bruker AXS GmbH*. X‐ray radiation was generated by a microfocus source IμS Mo by *Incoatec Gmb*H with HELIOS mirror optics and a single‐hole collimator by *Bruker AXS GmbH*. For the data collection, the programs *APEX 4 Suite* (v.2020.10‐0) with the integrated programs SAINT (integration) and SADABS (absorption correction) by *Bruker AXS GmbH* were used.

Using Olex2, the structures were solved with the ShelXT structure solution program using Intrinsic Phasing and refined with the ShelXL refinement package using Least Squares minimization. MicroGrippers from *MiTeGen* were used for mounting. For mass spectrometric evaluation a GC/EI‐MS system with a nominal‐resolution ISQ mass spectrometer coupled to a *Thermo Trace GC Ultra* oven and a direct probe controller from *Thermo Fischer Scientific* was used. The capillary column used was an OPTIMA (5 MS–0.25 μm; 30 m, 0.25 mm+10 m VS) from *Macherey‐Nagel*. Helium was used as the carrier gas. For high resolution mass spectrometry data a LTQ‐Orbitrap (Linear Trap Quadrupole Orbitrap) from *Thermo Fischer Scientific* coupled to a *Shimadzu* HPLC consisting of a CBM‐20A communication module, a SPD‐M30A UV detector, a CTO‐20AC column oven, a SIL‐30AC autosampler, a LC‐20ADXR pump system, and a DGU‐20A5R degasser unit were used. Elemental analyses were performed with the elemental analyzer *vario MICRO cube* from the company *Elementar* and the weighing of the substance quantities was done with the microbalance *Cubis MSE3.6P* from the company *Sartorius*. Optical rotations were measured with an *A. Krüss* Optical polarimeter operating on the sodium D‐line (589 nm) using a quartz glass cuvette (1 mL) and are reported as: [*α*]_*D*_^*T*^(concentration in g/100 mL, solvent). The melting point for compound 1 was obtained using a *Büchni* M‐560 melting point apparatus and the resulting value is non‐corrected.

**Compound** (*S*)‐**2**: (*S*)‐**7** (4.2 ml, 34 mmol, 1.0 equiv. *e.r*.: >99 : 1) was suspended portion wise in 95 % formic acid (15 ml, 0.36 mol, 10 equiv.) under ice cooling and 37 % formaldehyde solution (10 ml, 0.11 mol, 3.2 equiv.) was subsequently added portion wise. The reaction solution was then stirred for 6 h under reflux and then for 12 h at room temperature. Subsequently, the reaction solution was adjusted to a pH of 13 by adding 2 M sodium hydroxide solution. The aqueous phase was extracted with Et_2_O (3×50 ml) and the combined organic phases were dried over Na_2_SO_4_. After removal of the solvent, the residue was purified by “Kugelrohr”‐distillation (oven temperature: 50 °C, pressure: 0.7 mbar). The desired product (*S*)‐**2** was obtained as a colorless oil (isolated yield: 3.93 g, 26.3 mmol, 75.9 %). ^**1**^ **H NMR** (400.3 MHz, C_6_D_6_): *δ*=1.23 [d, 3H, ^3^ *J*_HH_=6.66 Hz; PhCHC*H*_3_], 2.08 [s, 6H; N(C*H*_3_)_2_], 3.07 (q, 1H, ^3^ *J*_HH_=6.66 Hz; PhC*H*), 7.08–7.12 (m, 1H; *H*_para_), 7.18–7.21 (m, 2H; *H*_ortho_), 7.31–7.33 (m, 2H; *H*_meta_). **{^1^H}^13^C NMR** (100.6 MHz, C_6_D_6_): *δ* =21.1 (1C; CH*C*H_3_), 43.7 [2C; CHN(*C*H_3_)_2_], 66.6 (1C; Ph*C*HN), 127.4 (1C; *C*_para_), 128.1 (2C; *C*_meta_), 128.9 (2C; *C*_ortho_), 145.9 (1C; N*C*_ipso_). **GC/EI‐MS**: *t*_R_=6.04 min [80 °C (1 min)–7 °C/min–170 °C (2.5 min)‐50 °C/min–250 °C (1 min)]; *m*/*z* (%): 149 (24.5) [M^+^], 134 (100) [(M−Me)^+^], 105 (47) [(M−NMe_2_)^+^], 91 (23), 77 (36) [(Ph)^+^], 72 (79) [(M−Ph)^+^]. **Specific rotation**: [*α*]_*D*_^*20*^=−65.02° mL g^−1^ dm^−1^ (C_6_H_6_, 9.02 mg/mL).

**Compound** (*S*,*S*_Si_/*R*_Si_)‐**3**: (*S*)‐**2** (2.00 g, 13.4 mmol, 1.0 equiv.) was dissolved in Et_2_O (80 ml). The solution was cooled to 0 °C and *t*‐Butyllithium (7.76 ml, 14.7 mmol, 1.9 M in *n*‐pentane, 1.1 equiv.) was dropped into solution. The yellowish solution was stirred for 1.5 h at 0 °C. Phenylmethyldimethoxysilane (3.17 g, 17.4 mmol, 1.3 equiv.) was then added to the solution and the solution was slowly thawed to room temperature with stirring and stirred for 24 h in total. The solids were separated and the solvent was removed in vacuum. After purification by “Kugelrohr”‐distillation at reduced pressure (140 °C, 0.25 mbar), the product (*S*,*S*_Si_/*R*_Si_)‐**3** was isolated as a colorless oil [isolated yield: 3.34 g, 11.2 mmol, 83 %, *d.r*. (*S*,*S*_Si_/*S*,R_Si_)=56 : 44]. ^**1**^ **H NMR** (400.2 MHz, C_6_D_6_): *δ*=D_maj_ 0.63, D_min_ 0.66 (s, 3H; SiC*H*_3_), D_min_ 1.04, D_maj_ 1.08 (d, 3H, ^3^ *J*_H,H_=6.48 Hz; NCHC*H*_3_), D_maj_ 1.76, D_min_ 1.81 [s, 6H; N(C*H*_3_)_2_], D_min_ 3.34, D_maj_ 3.36 (s, 3H; SiOC*H*_3_), D_min_ 3.52, D_maj_ 3.57 (q, 1H, ^3^ *J*_H,H_=6.48 Hz; NC*H*), 7.16–7.18 (m, 2H; C*H*_ortho_), 7.21–7.33 (m, 3H; C*H*_ar_), 7.54–7.59 (m, 2H; C*H*_meta_), 7.63 (d, 1H, ^n^ *J*_H,H_=7.70 Hz; C_meta_), 8.04–8.11 (m, 1H; C*H*_para_). **{^1^H}^13^C NMR** (100.6 MHz, C_6_D_6_): *δ*=D_maj_ −3.0, D_min_ −1.9 (1C; Si*C*H_3_), D_min_ 17.4, D_maj_ 18.9 (1C; NCH*C*H_3_), D_min_ 42.5, D_maj_ 42.8 [2C; N(*C*H_3_)_2_], D_maj_ 50.8, D_min_ 50.9 (1C; O*C*H_3_), D_min_ 63.7, D_maj_ 64.2 (1C; N*C*H), 126.7 (2C; *C*_ar_), 128.3 (2C; *C*_ortho_), 129.8 (1C; *C*_ar_), 130.9 (1C; *C*_ar_), 134.6 (2C; *C*_meta_), 136.9 (1C; *C*_ar_), 138.0 (1C; Si*C*_ipso_), 138.6 (1C; Si*C*_ipso_), 153.3 (1C; NCH*C*_ortho_). **{^1^H}^29^Si NMR** (79.5 MHz, C_6_D_6_): *δ*=D_min_ −6.3, D_maj_ −5.4 (1Si). **GC/EI‐MS**: *t*_R_=5.99 min [80 °C (1 min)–10 °C min−1–1–250 °C (5.5 min)]; *m*/*z* (%): 299 (1) [(M^+^)], 284 (17) [(M−CH_3_)^+^], 268 (3) [(M−OCH_3_)^+^], 252 (9) [(M−OCH_3_−CH_3_)^+^], 223 (7) [(M−Ph)^+^], 206 (100) [(M−Ph−CH_3_−H)^+^]. **Elemental analysis**: Calc.: C: 72.19 %, H: 8.41 %, N: 4.68 %. Found: C: 72.0 %, H: 8.4 %, N: 4.4 %.

**Compound** (*S*,*S*_Si_)‐**1**: (*S*,*S*_Si_/*R*_Si_)‐**3** (3.06 g, 10.3 mmol, 1.0 equiv.) was dissolved in Et_2_O (80 ml). 2 M HCl (80 ml, 160 mmol, 15.6 equiv.) was added to the solution and the emulsion was intensively stirred for 2 h. After that the reaction mixture was then cooled to 0 °C and adjusted to pH 13 with KOH. The phases were separated and the aqueous phase was extracted with Et_2_O (3×15 mL). The combined organic phases were dried with MgSO_4_ and the volume of the solution was concentrated to 30 ml. After storage at −30 °C for 12 h, the product (*S*,*S*_Si_)‐**1** was obtained in the form of colorless crystals [isolated yield: 1.07 g, 3.74 mmol, 36 % yield, *d.r*. (*S*,*S*_Si_/*R*_Si_): 97 : 3]. ^**1**^ **H NMR** (400.2 MHz, C_6_D_6_): *δ*=D_maj_ 0.63, D_min_ 0.66 (s, 3H; SiC*H*_3_), D_min_ 1.04, D_maj_ 1.08 (d, 3H, ^3^ *J*_H,H_=6.48 Hz; NCHC*H*_3_), D_maj_ 1.76, D_min_ 1.81 [s, 6H; N(C*H*_3_)_2_], D_min_ 3.34, D_maj_ 3.36 (s, 3H; SiOC*H*_3_), D_min_ 3.52, D_maj_ 3.57 (q, 1H, ^3^ *J*_H,H_=6.48 Hz; NC*H*), 7.16–7.18 (m, 2H; C*H*_ortho_), 7.21–7.33 (m, 3H; C*H*_ar_), 7.54–7.59 (m, 2H; C*H*_meta_), 7.63 (d, 1H, ^n^ *J*_H,H_=7.70 Hz; C_meta_), 8.04–8.11 (m, 1H; C*H*_para_). **{^1^H}^13^C NMR** (100.6 MHz, C_6_D_6_): *δ*=D_maj_ −3.0, D_min_ −1.9 (1C; Si*C*H_3_), D_min_ 17.4, D_maj_ 18.9 (1C; NCH*C*H_3_), D_min_ 42.5, D_maj_ 42.8 [2C; N(*C*H_3_)_2_], D_maj_ 50.8, D_min_ 50.9 (1C; O*C*H_3_), D_min_ 63.7, D_maj_ 64.2 (1C; N*C*H), 126.7 (2C; *C*_ar_), 128.3 (2C; *C*_ortho_), 129.8 (1C; *C*_ar_), 130.9 (1C; *C*_ar_), 134.6 (2C; *C*_meta_), 136.9 (1C; *C*_ar_), 138.0 (1C; Si*C*_ipso_), 138.6 (1C; Si*C*_ipso_), 153.3 (1C; NCH*C*_ortho_). **{^1^H}^29^Si NMR** (79.5 MHz, C_6_D_6_): *δ* =D_min_ −6.3, D_maj_ −5.4 (1Si). **GC/EI‐MS**: *t*_R_=5.99 min [80 °C (1 min)–10 °C min−1‐1–250 °C (5.5 min)]; *m*/*z* (%): 299 (1) [(M^+^)], 284 (17) [(M−CH_3_)^+^], 268 (3) [(M−OCH_3_)^+^], 252 (9) [(M−OCH_3_−CH_3_)^+^], 223 (7) [(M−Ph)^+^], 206 (100) [(M−Ph−CH_3_−H)^+^].

**Elemental analysis**: Calc.: C: 72.19 %, H: 8.41 %, N: 4.68 %. Found: C: 72.0 %, H: 8.4 %, N: 4.4 %.

**Compound** (*S*)‐**5**: (*S*,*S*_Si_)‐**1** (40.0 mg, 0.140 mmol, 1.0 equiv., *d.r*.≥99 : 1) was dissolved in Et_2_O (3.0 mL). Hexamethyldisilazane (0.03 mL, 0.154 mmol, 1.1 equiv.) was then added to the reaction solution. The solution was then stirred for 20 h. The solvent and volatiles were removed under vacuum and the siloxane (*S*)‐**5** was obtained as a colorless oil [isolated yield: 0.40 g, 0.11 mmol, 80 %, *d.r*. (*S*,*S*_Si_,*R*_Si_)≥99 : 1]. ^**1**^ **H NMR** (400.3 MHz, C_6_D_6_): *δ*=0.13 [s, 9H; Si(C*H*_3_)_3_], 0.69 (s, 3H; SiC*H*_3_), 1.14 (d, ^3^ *J*_HH_=6.4 Hz, 3H; NCHC*H*_3_), 1.86 [s, 6H; N(C*H*_3_)_3_], 3.50 (q, ^3^ *J*_HH_=6.4 Hz, 1H; NC*H*CH_3_), 7.15–7.17 (m, 2H; C*H*_ar_), 7.22 (m, 1H; C*H*_ar_), 7.32 (m, 1H; C*H*_ar_), 7.54–7.57 (m, 2H; C*H*_ar_), 7.74 (d, ^3^ *J*_HH_=7.7 Hz 1H; SiCC*H*_ar_), 7.95 (d, ^3^ *J*_HH_=8.6, 2H; C*H*_ar_). **{^1^H}^13^C NMR** (100.6 MHz, C_6_D_6_): *δ*=1.0 (1C; Si*C*H_3_), 2.5 [3C; Si(*C*H_3_)_3_], 21.0 (1C; NCH*C*H_3_), 43.4 [2C; N(*C*H_3_)_2_], 64.8 (1C; N*C*H), 126.7 (1C; *C*_ar_), 127.0 (1C; *C*_ar_), 128.3 (1C; *C*_ar_), 128.5 (1C; *C*_ar_) 129.8 (1C; *C*_ar_), 130.9 (1C; *C*_ar_), 134.3 (2C; *C*_ar_), 136.0 (1C; Si*C*_ipso_), 136.2 (1C; *C*_ar_), 140.6 (1C; Si*C*_ipso_), 153.3 (1C; NCH*C*_ortho_). **{^1^H}^29^Si NMR** (79.5 MHz, C_6_D_6_): *δ* =D_min_‐14.8, D_maj_ −13.6 (1Si; PhMe*Si*O), D_min_ −8.4, D_maj_ −8.9 (1Si, *Si*Me_3_). **GC/EI‐MS** *t*_R_=6.00 min [80 °C (1 min)–20 °C/min–290 °C (2 min)]; *m*/*z*: 342 (10) [(M−Me)^+^], 297 (14) {[M−H_3_CHN(CH_3_)_2_]^+^}, 264 (100) [(M−Ph−Me)^+^], 209 (21) {[PhMeSiOSi(CH_3_)_3_]^+^}, 72 (41) {[Si(CH_3_)_3_]^+^}, 59 (1) {[CH_2_N(CH_3_)_2_]^+^}. *m*/*z*: 344 (10) [(^18^M−Me)^+^], 299 (14) {[^18^M−H_3_CHN(CH_3_)_2_]^+^}, 266 (100) [(^18^M−Ph−Me)^+^], 211 (4) {[PhMeSi^18^OSi(CH_3_)_3_]^+^}, 72 (40) {[Si(CH_3_)_3_]^+^}, 59 (1) {[CH_2_N(CH_3_)_2_]^+^}. **LC/HR‐MS**: *t*_R_=6.23 min [0.3 mL/min H_2_O/MeCN (+ 0.1 % MeCOOH): 95 : 5 (2 min)–80 : 20 (*Δt*=3 min, 3 min)–0 : 100 (*Δt*=12 min, 8 min)–95 : 5 (*Δt*=4 min, 2 min)]; 358.20225 *m*/*z* {C_20_H_32_ONSi_2_ [M+H]^+^, (*Δ*=1.552 ppm)} 360.20618 *m*/*z* {C_20_H_32_^18^ONSi_2_ [M+H]^+^, (*Δ*=0.666 ppm)} **Specific rotation**: [*α*]_*D*_^*20*^=+7.338° mL g^−1^ dm^−1^ (C_6_H_6_, 5.6 mg/mL).

**Elemental analysis**: Calc: C: 67.17 %, H: 8.74 %, N: 3.92 %. Found: C: 67.3 %, H: 8.7 %, N: 4.0 %.

**Compound** (*S*,*S*,*S*_Si_,*S*_Si_)‐**6**: (*S*,*S*_Si_)‐**1** (0.04 g, 0.15 mmol, 1.0 equiv., *d.r*.≥99 : 1) was dissolved in 1 mL acetone. ZnBr_2_ (0.02 g, 0.08 mmol, 0.5 equiv.) was dissolved in 1 mL acetone and added to the silanol solution. After a few days, the product (*S*,*S*,*S*_Si_,*S*_Si_)‐**6** crystallized from acetone in the form of colorless blocks in space group *P*2_1_2_1_2_1_ (0.06 g, 0.07 mmol, 97 % yield). **Yield**: 0.06 g, 0.07 mmol (96.7 %, in relation of 2x C_34_H_46_Br_2_N_2_O_2_Si_2_Zn⋅C_3_H_6_O) **Elemental analysis**: Calc.: C: 52.03 %, H: 6.14 %, N: 3.28 %. Found: C: 52.3 %, H: 6.2 %, N: 3.4 %.

## Conflict of interest

The authors declare no conflict of interest.

1

## Supporting information

As a service to our authors and readers, this journal provides supporting information supplied by the authors. Such materials are peer reviewed and may be re‐organized for online delivery, but are not copy‐edited or typeset. Technical support issues arising from supporting information (other than missing files) should be addressed to the authors.

Supporting Information

## Data Availability

The data that support the findings of this study are available in the supplementary material of this article.
