# Supplementary material for: A Silicon‐Stereogenic Silanol ‐ 18O‐Isotope Labeling and Stereogenic Probe Reveals Hidden Stereospecific Water Exchange Reaction
Source: Chemistry. 2022 Nov 10;29(1):e202202935. doi: 10.1002/chem.202202935 (PMC10100314; doi:10.1002/chem.202202935)
Supplement: Supplementary file 1 — Supporting Information [file CHEM-29-0-s001.pdf]

Chemistry–A European Journal

Supporting Information

A Silicon-Stereogenic Silanol - ^{18}O-Isotope Labeling and Stereogenic Probe Reveals Hidden Stereospecific Water Exchange Reaction

Felix Langenohl, Jonas Rösler, Sebastian Zühlke,* Jan-Lukas Kirchhoff, and Carsten Strohmann*

Table of contents

1. General remarks 4

2. Chemicals 6

3. Experimental procedures 6

3.1. Synthesis of amine (*S*)-2 6

Synthesis of amine (*S*/*R*)-2: 7

3.2. (*S*,S_{Si}/R_{Si})-phenylmethylmethoxysilane 3 8

3.3. Synthesis of (*S*,S_{Si})-phenylmethylsilanol 1 10

Direct synthesis of phenylmethylsilanol 1 from amine (*S*)-2: 11

3.4. Synthesis of (*S*)-siloxane 5 12

3.5. Synthesis of siloxane 5 via lithium siloxide: 13

3.6. Synthesis of (*S*,*S*)-siloxane 8 14

3.7. Synthesis and crystallization of zinc complex (*S*,*S*,S_{Si},S_{Si})-6 16

4. Single crystal X-ray diffraction analysis 17

4.1. Crystal structure of silanol (*S*,S_{Si})-1 17

4.2. Crystal structure of zinc complex 6 20

4.3. Diastereomeric ratios and absolute configuration 23

4.4. Verification of diastereomeric ratios 23

5. NMR studies with silanol 1 25

5.1. Silanol 1 in benzene-d_6 25

5.2. Silanol 1 in Et_2O 27

5.3. Silanol 1 in THF 29

6. NMR spectra of compounds 31

6.1. NMR spectra of amine 2 31

6.2. NMR spectra of methoxysilane 3 32

6.3. NMR spectra of silanol (S,S_{Si})-1 34

6.4. NMR Spectra of siloxane 5 35

7. Mass spectrometric studies 37

7.1. Experiment with H_2O-^{18}O 37

7.2. Calibration: 38

7.3. Additional experiment: 39

7.4. Values of the GC/EI-MS experiment: silanol 1 in THF 40

8. IR studies 43

8.1. IR spectrum of silanol (S,S_{Si})-1 43

8.2. IR spectrum of zinc complex (S,S,S_{Si},S_{Si})-6 44

9. References 44

1. General remarks

All reactions with oxygen- and moisture-sensitive compounds were performed under an atmosphere of argon with SCHLENK-techniques in dried solvents. The solvents were purified and dried by distillation over sodium and storage under argon atmosphere. Commercially available reagents were used without further purification except for phenylmethyl-dimethoxysilane, which was distilled and stored over molsieve (4 Å) in argon atmosphere.

The **NMR spectra** were measured on a Bruker Avance III HD NanoBay - 400 MHz, 500 MHz Bruker Avance NEO, 600 MHz Bruker Avance III HD and 500 MHz Agilent Technologies DD2 spectrometer at 25 °C. Chemical shifts (δ in ppm) are referred to tetramethylsilane (TMS), with the deuterium signal of the solvent serving as internal lock and the residual solvent signal as additional reference [^{1}H-NMR δ(C_6D_5H) = 7.16 ppm). Signals were assigned with the help of HSQC experiments. For the multiplicities following abbreviations were used: s = singlet, br = broad signal, d = doublet, q = quartet. In diastereomeric associations, diastereomeric signals were assigned to the major diastereomer (D_{maj}) and the minor diastereomer (D_{min}) if distinguishable. For siloxane **8** the observed diastereomers were labeled as D_1, D_2 and D_3. For spectra measured in non-deutered solvents, a capillary with C_6D_6 was added and served as an internal standard. ^{13}C-NMR Spectra were measured with broadband decoupling and referred to the signal of the solvent [{^{1}H}^{13}C-NMR δ(C_6D_6) = 128.39 ppm]. {^{1}H}^{29}Si-NMR spectra were as inverted gated with TMS as external standard.

Single crystal X-ray diffraction for compounds **1** and **6** were conducted on a *Bruker D8 Venture* four-circle diffractometer by *Bruker AXS GmbH* using a PHOTON II CPAD detector by *Bruker AXS GmbH*. X-ray radiation was generated by a microfocus source IμS Mo by *Incoatec Gmb*H with HELIOS mirror optics and a single-hole collimator by *Bruker AXS GmbH*. For the data collection, the programs *APEX 4 Suite* (v.2020.10-0) with the integrated programs SAINT (integration) and SADABS (absorption correction) by *Bruker AXS GmbH* were used.[1]

Using Olex2,[2] the structures were solved with the ShelXT[3] structure solution program using Intrinsic Phasing and refined with the ShelXL[4] refinement package using Least Squares minimization. MicroGrippers from *MiTeGen* were used for mounting.[5]

For **mass spectrometric evaluation** a GC/EI-MS system with a nominal-resolution ISQ mass spectrometer coupled to a *Thermo Trace GC Ultra* oven and a direct probe controller from *Thermo Fischer Scientific* was used. The capillary column used was an OPTIMA (5

MS – 0.25 µm; 30 m, 0.25 mm + 10 m VS) from *Macherey-Nagel*. Helium was used as the carrier gas.

For high resolution mass spectrometry data a LTQ-Orbitrap (Linear Trap Quadrupole Orbitrap) from *Thermo Fischer Scientific* coupled to a *Shimadzu* HPLC consisting of a CBM-20A communication module, a SPD-M30A UV detector, a CTO-20AC column oven, a SIL-30AC autosampler, a LC-20ADXR pump system, and a DGU-20A5R degasser unit were used.

Elemental analyses were performed with the elemental analyzer *vario MICRO cube* from the company *Elementar* and the weighing of the substance quantities was done with the microbalance *Cubis MSE3.6P* from the company *Sartorius*.

Specific rotations were measured with an *A. Krüss* Optical polarimeter operating on the sodium D-line (589 nm) using a quartz glass cuvette (1 mL) and are reported as: $[\alpha]_D^T$(concentration in g/100 mL, solvent).

The **melting point** for compound **1** was obtained using a *Büchni* M-560 melting point apparatus and the resulting value is non-corrected.

2. Chemicals

compound	Supplier	purity
THF	*Fisher chemicals*	99,8%
Et_2O	*Köster & Bömcke Service GmbH*	≥98.0%
acetone	*Köster & Bömcke Service GmbH*	≥97.0%
benzene-*d*$_6$	*Eurisotop*	99.5%
H_2O-^{18}O	*J&K-scientific*	97.0%
amine (*S*/*R*)-**7**	*Merck*	99.0%
amine (*S*)-**7** (*e.r.* >99:1)	*Acros*	98.0%
formaldehyde	*ABCR*	36% in water
tert-butyllithium	*Acros*	(1.9 M in *n*-pentane)
phenylmethyldimethoxysilane	*ABCR*	97%
hexamethyldisilazane	*Fluka*	≥98.0%
trimethylsilyl chloride	*Acros*	98.0%
$ZnBr_2$ (anhydrous)	*ABCR*	≥98.0%

3. Experimental procedures

3.1. Synthesis of amine (*S*)-2

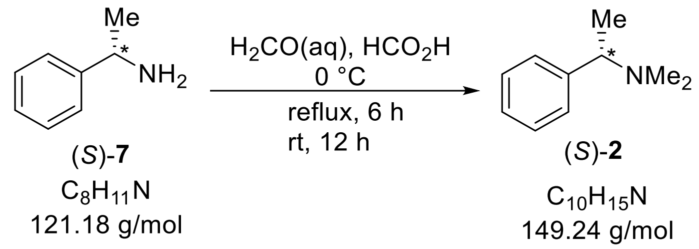

Methylbenzylamine (*S*)-**7** (4.2 ml, 34 mmol, 1.0 eq. *e.r.* >99:1) was suspended portion wise in 95% formic acid (15 ml, 0.36 mol, 10 eq.) under ice cooling and 37% formaldehyde solution (10 ml, 0.11 mol, 3.2 eq.) was subsequently added portion wise. The reaction solution was then stirred for 6 h under reflux and then for 12 h at room temperature. Subsequently, the reaction solution was adjusted to a pH of 13 by adding 2 M sodium hydroxide solution. The aqueous phase was extracted with Et_2O (3 x 50 ml) and the combined organic phases were dried over Na_2SO_4. After removal of the solvent, the residue was purified by “Kugelrohr”-distillation (oven temperature: 50 °C, pressure: 0.7 mbar). The desired product was obtained as a colorless oil (isolated yield: 3.93 g, 26.3 mmol, 75.9%). The analytic data is consistent with those from the literature.[6]

Boiling point: ≈ 45 °C (0.7 mbar)

^{1}H-NMR (400.3 MHz, C_6D_6): δ = 1.23 [d, 3H, $^3J_{HH}$ = 6.66 Hz; PhCHCH_3], 2.08 [s, 6H; N(CH_3)$_2$], 3.07 (q, 1H, $^3J_{HH}$ = 6.66 Hz; PhCH), 7.08-7.12 (m, 1H; H_{para}), 7.18-7.21 (m, 2H; H_{ortho}), 7.31-7.33 (m, 2H; H_{meta}).

{^{1}H}^{13}C-NMR (100.6 MHz, C_6D_6): δ = 21.1 (1C; CHCH$_3$), 43.7 [2C; CHN(CH$_3$)$_2$], 66.6 (1C; PhCHN), 127.4 (1C; C_{para}), 128.1 (2C; C_{meta}), 128.9 (2C; C_{ortho}), 145.9 (1C; NC_{ipso}).

GC/EI-MS: t_R = 6.04 min [80 °C (1 min) – 7°C/min – 170 °C (2.5 min) – 50 °C/min – 250 °C (1 min)]; *m/z* (%): 149 (24.5) [M$^+$], 134 (100) [(M – Me)$^+$], 105 (47) [(M – NMe$_2$)$^+$], 91 (23), 77 (36) [(Ph)$^+$], 72 (79) [(M-Ph)$^+$].

Specific rotation: $[\alpha]_D^{20}$ = –65.02° mL g^{-1} dm^{-1} (C_6H_6, 9.02 mg/mL).

Synthesis of amine (*S*/*R*)-2:

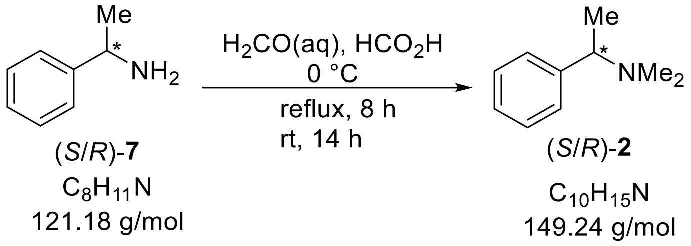

(*S*/*R*)-**7**
$C_8H_{11}N$
121.18 g/mol

(*S*/*R*)-**2**
$C_{10}H_{15}N$
149.24 g/mol

Methylbenzylamine (*S*/*R*)-**7** (31.9 mL, 0.24 mol, 1.0 eq. *e.r.* ~ 50:50) was suspended portion wise in 95% formic acid (90 mL, 1.88 mol, 7.5 eq.) under ice cooling and 37% formaldehyde solution (90 mL, 1.11 mol, 4.5 eq.) was subsequently added portion wise. The reaction solution was then stirred for 8 h under reflux and then for 14 h at room temperature. Subsequently, the reaction solution was adjusted to a pH of 13 by adding 2 M sodium hydroxide solution. The aqueous phase was extracted with Et_2O (3 x 150 ml) and the combined organic phases were dried over Na_2SO_4. After removal of the solvent, the residue was purified by distillation (head temperature: 45 °C, pressure: 0.7 mbar). The desired product was obtained as a colorless oil (isolated yield: 27.2 g, 0.18 mol, 74%).

3.2. (*S*,S_{Si}/R_{Si})-phenylmethylmethoxysilane 3

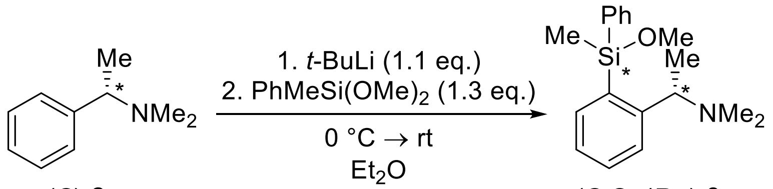

(*S*)-**2** — $C_{10}H_{15}N$, 149.12 g/mol

(*S*,S_{Si}/R_{Si})-**3** — $C_{18}H_{25}NOSi$, 299.17 g/mol

Amine (*S*)-**2** (2.00 g, 13.4 mmol, 1.0 eq.) was dissolved in Et_2O (80 ml). The solution was cooled to 0 °C and *t*-Butyllithium (7.76 ml, 14.7 mmol, 1.9 M in *n*-pentane, 1.1 eq.) was dropped into solution. The yellowish solution was stirred for 1.5 h at 0 °C. Phenylmethyldimethoxysilane (3.17 g, 17.4 mmol, 1.3 eq.) was then added to the solution and the solution was slowly thawed to room temperature with stirring and stirred for 24 h in total. The solids were separated and the solvent was removed in vacuum. After purification by "KUGELROHR"-distillation at reduced pressure (140 °C, 0.25 mbar), the product was isolated as a colorless oil [isolated yield: 3.34 g, 11.2 mmol, 83%, *d.r.*(*S*,S_{Si}/*S*,R_{Si}) = 56:44].

Boiling point: 140 °C (0.25 mbar)

^{1}H-NMR (400.2 MHz, C_6D_6): δ = D_{maj} 0.63, D_{min} 0.66 (s, 3H; SiCH_3), D_{min} 1.04, D_{maj} 1.08 (d, 3H, $^3J_{H,H}$= 6.48 Hz; NCHCH_3), D_{maj} 1.76, D_{min} 1.81 [s, 6H; N(CH_3)$_2$], D_{min} 3.34, D_{maj} 3.36 (s, 3H; SiOCH_3), D_{min} 3.52, D_{maj} 3.57 (q, 1H, $^3J_{H,H}$ = 6.48 Hz; NC*H*), 7.16-7.18 (m, 2H; CH_{ortho}), 7.21-7.33 (m, 3H; CH_{ar}), 7.54-7.59 (m, 2H; CH_{meta}), 7.63 (d, 1H, $^nJ_{H,H}$= 7.70 Hz; C_{meta}), 8.04-8.11 (m, 1H; CH_{para}).

{^{1}H}^{13}C-NMR (100.6 MHz, C_6D_6): δ = D_{maj} –3.0, D_{min} –1.9 (1C; SiCH_3), D_{min} 17.4, D_{maj} 18.9 (1C; NCHCH_3), D_{min} 42.5, D_{maj} 42.8 [2C; N(CH_3)$_2$], D_{maj} 50.8, D_{min} 50.9 (1C; OCH_3), D_{min} 63.7, D_{maj} 64.2 (1C; N*C*H), 126.7 (2C; C_{ar}), 128.3 (2C; C_{ortho}), 129.8 (1C; C_{ar}), 130.9 (1C; C_{ar}), 134.6 (2C; C_{meta}), 136.9 (1C; C_{ar}), 138.0 (1C; SiC_{ipso}), 138.6 (1C; SiC_{ipso}), 153.3 (1C; NCHC_{ortho}).

{^{1}H}^{29}Si-NMR (79.5 MHz, C_6D_6): δ = D_{min} –6.3, D_{maj} –5.4 (1Si).

GC/EI-MS: t_R = 5.99 min [80 °C (1 min) – 10 °C/min-1 – 250 °C (5.5 min)]; *m*/*z* (%): 299 (1) [(M^+)], 284 (17) [($M – CH_3)^+$], 268 (3) [($M – OCH_3)^+$], 252 (9) [($M – OCH_3 – CH_3)^+$], 223 (7) [($M – Ph)^+$], 206 (100) [($M – Ph – CH_3 – H)^+$].

**Elemental analysis:**	Calc.:	C: 72.19%	H: 8.41%	N: 4.68%
	Found:	C: 72.0%	H: 8.4%	N: 4.4%

Synthesis of phenylmethoxysilane (S/R)-3:

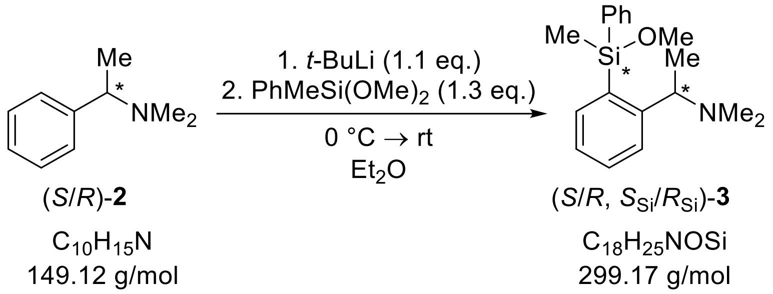

Amine (*S*/*R*)-**2** (1.00 g, 6.71 mmol, 1.0 eq.) was dissolved in Et_2O (50 ml). The solution was cooled to 0 °C and *t*-Butyllithium (3.89 mL, 7.38 mmol, 1.9 M in *n*-pentane, 1.1 eq.) was dropped into solution. The yellowish solution was stirred for 1.5 h at 0 °C. Phenylmethyldimethoxysilane (1.58 g, 8.72 mmol, 1.3 eq.) was then added to the solution and the solution was slowly thawed to room temperature with stirring and stirred for 24 h in total. The solids were separated and the solvent was removed in vacuum. After purification by “KUGELROHR”-distillation at reduced pressure (140 °C, 0.25 mbar), the product was isolated as a colorless oil [isolated yield: 1.09 g, 3.63 mmol, 54% yield; *d.r.* (S,S_{Si}/R,R_{Si}: R,S_{Si}/S,R_{Si}) = 54:46].

3.3. Synthesis of (S,S_{Si})-phenylmethylsilanol 1

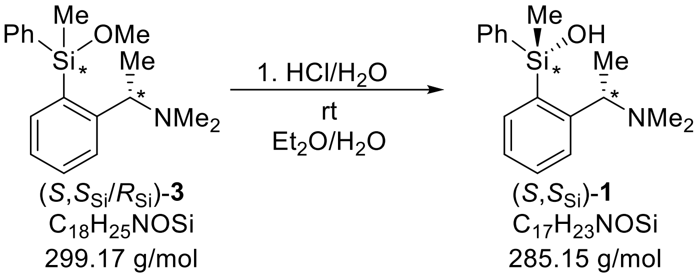

(S,S_{Si}/R_{Si})-phenylmethylmethoxysilane **3** (3.06 g, 10.3 mmol, 1.0 eq.) was dissolved in Et_2O (80 ml). 2 M HCl (80 ml, 160 mmol, 15.6 eq.) was added to the solution and the emulsion was intensively stirred for 2 h. After that the reaction mixture was then cooled to 0 °C and adjusted to pH 13 with KOH. The phases were separated and the aqueous phase was extracted with Et_2O (3 x 15 mL). The combined organic phases were dried with $MgSO_4$ and the volume of the solution was concentrated to 30 ml. After storage at –30 °C for 12 h, the product was obtained in the form of colorless crystals [isolated yield: 1.07 g, 3.74 mmol, 36% yield, *d.r.* (S,S_{Si}/R_{Si}): 97:3].

Melting point: 113.2 °C (1 °C/min)

^{1}H-NMR (400.2 MHz, C_6D_6): δ = D_{maj} 0.75, D_{min} 0.76 (s, 3H; SiCH_3), 0.98-1.03 (br, 3H; NCHCH_3), D_{maj} 1.84, D_{min} 1.87 [s, 6H; N(CH_3)$_2$], 3.48-3.73 (br, 1H; NCH), 7.02 (d, 1H, $^3J_{H,H}$ = 7.34 Hz; CH_{ortho}), 7.09-7.13 (m, 2H; CH_{ortho}), 7.18-7.27 (m, 3H; CH_{ar}), 7.67-7.72 (m, 1H; CH_{para}), 7.78-7.83 (m, 2H; CH_{meta}), 8.65-9.35 (br, 1H; SiOH).

{^{1}H}^{13}C-NMR (100.6 MHz, C_6D_6): δ = D_{maj} 1.1, D_{min} 2.4 (1C; SiCH$_3$), 15.9 (1C; NCHCH$_3$), 41.0 [2C; N(CH$_3$)$_2$], D_{maj} 66.3, D_{min} 66.6 (1C; NCH), 127.3 (1C; C_{ar}), 128.3 (1C; C_{ar}), 128.5 (1C; C_{ar}), 129.8 (2C; C_{ortho}), 134.6 (2C; C_{meta}), 136.9 (1C; C_{ar}), 137.5 (1C; SiC_{ipso}), 139.7 (1C; SiC_{ipso}), 141.7 (1C; C_{para}), 149.3 (1C; NCHC_{ortho}).

{^{1}H}^{29}Si-NMR (79.5 MHz, C_6D_6): δ = D_{min} –6.1, D_{maj} –5.2 (1Si).

LC/HR-MS: t_R = 10.63 [0.3 mL/min H_2O/MeCN (+ 0.1% MeCOOH): 95:5 (2 min) – 80:20 (Δt = 3 min, 3 min) – 0:100 (Δt =12 min, 8 min) – 95:5 (Δt = 4 min, 2 min)]; 286.16227 *m*/*z* {$C_{17}H_{24}ONSi$ [M + H]$^+$, (Δ = 0.372 ppm)}

Specific rotation: $[\alpha]_D^{20}$ = +1.045° mL g^{-1} dm^{-1} (C_6H_6, 6.86 mg/mL).

**Elemental analysis:**	Calc:	C: 71.53%	H: 8.12%	N: 4.91%
	Found:	C: 71.4%	H: 8.2%	N: 4.8%

Direct synthesis of phenylmethylsilanol 1 from amine (*S*)-2:

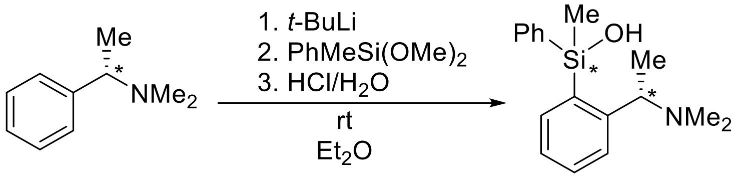

(*S*)-**2**
$C_{10}H_{15}N$
149.12 g/mol

(*S*,S_{Si}/R_{Si})-**1**
$C_{17}H_{23}NOSi$
285.15 g/mol

Amine (*S*)-**2** (1.50 g, 10.1 mmol, 1.0 eq.) was dissolved in Et_2O (50 ml). The solution was cooled to 0 °C and *t*-Butyllithium (5.82 mL, 11.1 mmol, 1.9 M in *n*-pentane, 1.1 eq.) was dropped into solution. The yellowish turbid solution was stirred for 2 h at 0 °C. Phenyl-methyldimethoxysilane (2.37 mL, 2.38 mmol, 1.3 eq.) was then added to the solution and the solution was slowly thawed to room temperature with stirring and stirred for 18 h in total. The reaction was terminated by addition of H_2O and adjusted to pH 2 with 2 M HCl. The phases were separated and the organic phase was extracted with 0.2 M HCl (3 x 10 mL). The combined aqueous phases were adjusted to pH 13 with KOH and extracted with Et_2O (3 x 15 mL). The combined organic phases were dried with Na_2SO_4. The solvent was removed on the rotary evaporator (isolated yield: 2.36 g, 8.27 mmol, 82% yield).

Synthesis of (*S*/*R*)-phenylmethoxysilanol 1:

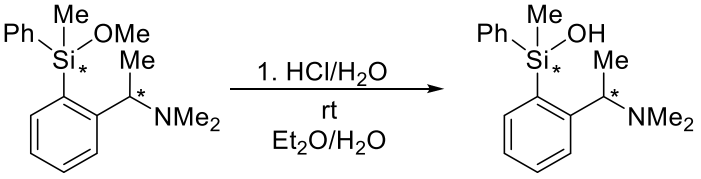

(*S*/*R*,S_{Si}/R_{Si})-**3**
$C_{18}H_{25}NOSi$
299.17 g/mol

(*S*/*R*,S_{Si}/R_{Si})-**1**
$C_{17}H_{23}NOSi$
285.15 g/mol

(*S*/*R*,S_{Si}/R_{Si})-phenylmethylmethoxysilane **3** (0.06 g, 0.20 mmol, 1.0 eq.) was dissolved in Et_2O (5 ml). 2 M HCl (2.0 mL, 1.00 mmoL, 5.0 eq.) was added to the solution and the emulsion was intensively stirred for 2 h. After that the reaction mixture was then cooled to 0 °C and adjusted to pH 13 with KOH. The phases were separated and the aqueous phase was extracted with Et_2O (3 x 5 mL). The combined organic phases were dried with $MgSO_4$

and the solvent was removed in vacuum. The product was obtained in the form of a colorless oil [isolated yield: 0.05 g, 0.19 mmol, *d.r.* (S,S_{Si}/R,R_{Si} : R,S_{Si}/S,R_{Si}) = 56:44].

3.4. Synthesis of (*S*)-siloxane 5

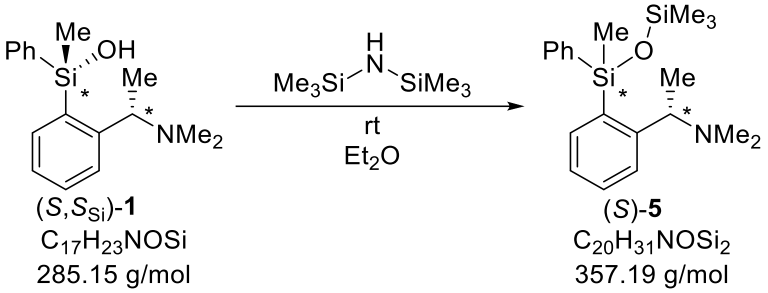

Silanol (S,S_{Si})-**1** (40.0 mg, 0.140 mmol, 1.0 eq., *d.r.* ≥ 99:1) was dissolved in Et_2O (3.0 mL). Hexamethyldisilazane (0.03 mL, 0.154 mmol, 1.1 eq.) was then added to the reaction solution. The solution was then stirred for 20 h. The solvent and volatiles were removed under vacuum and the siloxane (*S*)-**5** was obtained as a colorless oil [isolated yield: 0.40 g, 0.11 mmol, 80%, *d.r.* (S,S_{Si},R_{Si}) ≥ 99:1].

^{1}H-NMR (400.3 MHz, C_6D_6): δ = 0.13 [s, 9H; Si(C*H*$_3$)$_3$], 0.69 (s, 3H; SiC*H*$_3$), 1.14 (d, $^3J_{HH}$ = 6.4 Hz, 3H; NCHC*H*$_3$), 1.86 [s, 6H; N(C*H*$_3$)$_3$], 3.50 (q, $^3J_{HH}$ = 6.4 Hz, 1H; NC*H*CH$_3$), 7.15–7.17 (m, 2H; C*H*$_{ar}$), 7.22 (m, 1H; C*H*$_{ar}$), 7.32 (m, 1H; C*H*$_{ar}$), 7.54–7.57 (m, 2H; C*H*$_{ar}$), 7.74 (d, $^3J_{HH}$ = 7.7 Hz 1H; SiCC*H*$_{ar}$), 7.95 (d, $^3J_{HH}$ = 8.6, 2H; C*H*$_{ar}$).

{^{1}H}^{13}C-NMR (100.6 MHz, C_6D_6): δ = 1.0 (1C; Si*C*H$_3$), 2.5 [3C; Si(*C*H$_3$)$_3$], 21.0 (1C; NCH*C*H$_3$), 43.4 [2C; N(*C*H$_3$)$_2$], 64.8 (1C; N*C*H), 126.7 (1C; *C*$_{ar}$), 127.0 (1C; *C*$_{ar}$), 128.3 (1C; *C*$_{ar}$), 128.5 (1C; *C*$_{ar}$) 129.8 (1C; *C*$_{ar}$), 130.9 (1C; *C*$_{ar}$), 134.3 (2C; *C*$_{ar}$), 136.0 (1C; Si*C*$_{ipso}$), 136.2 (1C; *C*$_{ar}$), 140.6 (1C; Si*C*$_{ipso}$), 153.3 (1C; NCH*C*$_{ortho}$).

{^{1}H}^{29}Si-NMR (79.5 MHz, C_6D_6): δ = D_{min}–14.8, D_{maj} –13.6 (1Si; PhMe*Si*O), D_{min} –8.4, D_{maj} –8.9 (1Si, *Si*Me$_3$).

GC/EI-MS t_R = 6.00 min [80 °C (1 min) – 20 °C/min – 290 °C (2 min)]; *m*/*z*: 342 (10) [(M – Me)$^+$], 297 (14) {[M – H$_3$CHN(CH$_3$)$_2$]$^+$}, 264 (100) [(M – Ph – Me)$^+$], 209 (21) {[PhMeSiOSi(CH$_3$)$_3$]$^+$}, 72 (41) {[Si(CH$_3$)$_3$]$^+$}, 59 (1) {[CH$_2$N(CH$_3$)$_2$]$^+$}.

m/z: 344 (10) [(^{18}M – Me)$^+$], 299 (14) {[^{18}M – $H_3CHN(CH_3)_2$]$^+$}, 266 (100) [(^{18}M – Ph – Me)$^+$], 211 (4) {[PhMeSi^{18}OSi$(CH_3)_3$]$^+$}, 72 (40) {[Si$(CH_3)_3$]$^+$}, 59 (1) {[$CH_2N(CH_3)_2$]$^+$}.

LC/HR-MS: t_R = 6.23 min [0.3 mL/min H_2O/MeCN (+ 0.1% MeCOOH): 95:5 (2 min) – 80:20 (Δt = 3 min, 3 min) – 0:100 (Δt =12 min, 8 min) – 95:5 (Δt =4 min, 2 min)];

358.20225 *m/z* {$C_{20}H_{32}ONSi_2$ [M + H]$^+$, (Δ = 1.552 ppm)}

360.20618 *m/z* {$C_{20}H_{32}{}^{18}ONSi_2$ [M + H]$^+$, (Δ = 0.666 ppm)}

Specific rotation: $[\alpha]_D^{20}$ = +7.338° mL g^{-1} dm^{-1} (C_6H_6, 5.6 mg/mL).

Elemental analysis:

Calc:	C: 67.17%	H: 8.74%	N: 3.92%
Found:	C: 67.3%	H: 8.7%	N: 4.0%

3.5. Synthesis of siloxane 5 via lithium siloxide:

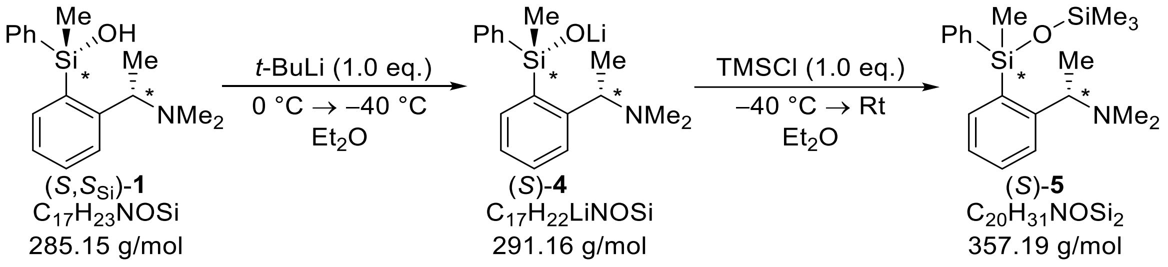

Silanol (S,S_{Si})-**1** (55.3 mg, 0.17 mmol, 1.0 eq. *d.r.* = 99:1) was dissolved in Et_2O (1.2 mL). Then *t*-Butyllithium (0.09 mL, 0.17 mmol, 1.9 M in *n*-pentane, 1.0 eq.) was added to the reaction solution at 0 °C. The solution was then stirred for 30 min. At –40 °C, trimethylsilyl chloride (0.02 mL, 0.17 mmol, 1.0 eq.) was added. The solution was slowly thawed to room temperature and continued stirring for one day. After addition of water (2 mL), the pH was adjusted to pH = 13 at 0 °C with KOH. The organic phase was extracted (3 x 30 mL) and the solution was dried over $MgSO_4$. After filtration, the was removed under vacuum. The siloxane (*S*)-**5** was obtained diastereoselectively (yield: 2.36 g. 8.27 mmol, 82%; *d.r.* = 99:1).

Lithium siloxide (*S*)-**4** was not isolated. The formation of the component was observed by a slight yellow coloration of the solution and confirmed by NMR spectroscopy. Only ^{29}Si- and

^{7}Li-NMR spectra were evaluated, since the ^{1}H- and ^{13}C-NMR spectra show large amounts of pentane as the main component in the solution, which was introduced via the *t*-butyllithium solution used. the used *t*-butyllithium solution overlapped with signals from ^{1}H and ^{13}C-NMR spectra.

{^{1}H}^{29}Si-NMR (79.5 MHz, C_6D_6): δ = D$_{maj}$ –12.8 (1Si; PhMe*Si*OLi).

{^{1}H}^{7}Li-NMR (155.6 MHz, C_6D_6): δ = D$_{maj}$ 1.44 (1Si; PhMeSiO*Li*).

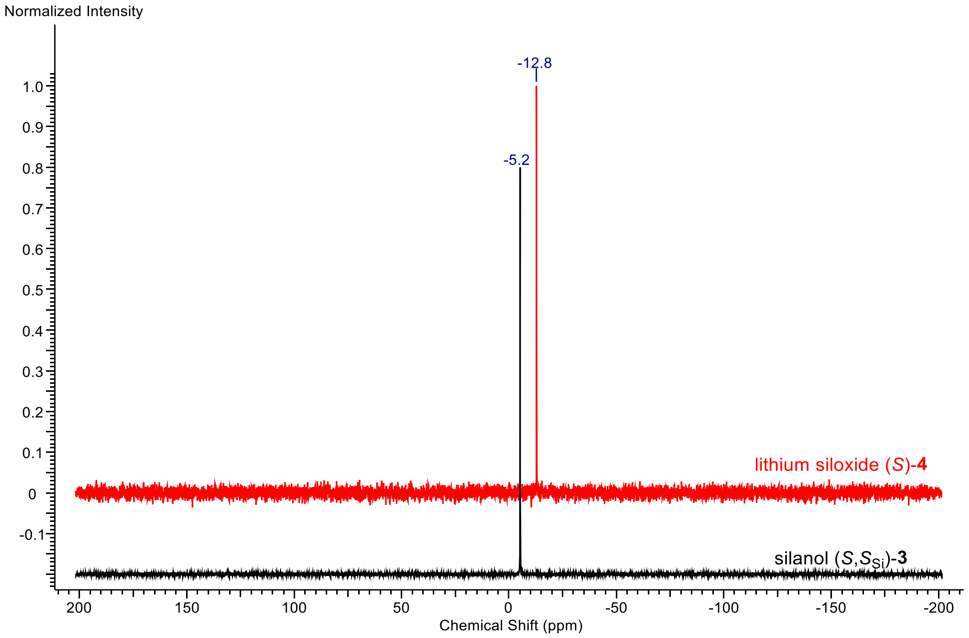

Figure S1: Signal shift on the ^{29}Si-NMR-spectra during the formation of lithium siloxide (*S*)-**4** from silanol (*S*,*S*$_{Si}$)-**1**.

3.6. Synthesis of (*S*,*S*)-siloxane 8

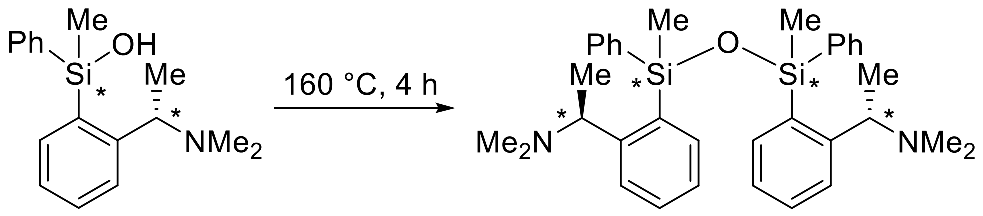

(*S*,*S*$_{Si}$/R$_{Si}$)-**1** (*S*,*S*,*S*$_{Si}$/R$_{Si}$,*S*$_{Si}$/R$_{Si}$)-**8**

$C_{17}H_{23}NOSi$ 285.15 g/mol

$C_{34}H_{44}N_2OSi_2$ 552.30 g/mol

Phenylmethylsilanol (S,S_{Si}/R_{Si})-**1** (0.21 g, 0.73 mmol, 2.0 eq.) was heated for 4 h (160 °C). The product was obtained as a high viscos colorless oil (0.16 g crude product yield, mixture of silanol **1** and siloxane **8**).

^{1}H-NMR (400.2 MHz, C_6D_6): δ = D_1 0.67, 0.71, D_2 0.68, D_3 0.72 (s, 6H; SiCH_3), 0.95-0.99 (m, 6H; NCHCH_3), D_2 1.72, D_1 1.75, 1.80, D_3 1.81 [s, 12H; N(CH_3)$_2$], 3.42-3.50 (m, 2H; 2 NCH), 7.09-7.16 (m, 5H; CH_{ar}), 7.19-7.27 (m, 4H; CH_{ar}), 7.28-7.33 (m, 2H; CH_{ar}), 7.50-7.57 (m, 4H; CH_{ar}), 7.59-7.62 (m, 1H; CH_{ar}), 8.16-8.24 (m, 2H; CH_{ar}).

{^{1}H}^{13}C-NMR (100.6 MHz, C_6D_6): δ = $D_{2/3}$ 0.8, D_1 0.9, 1.63, $D_{2/3}$ 1.70 (2C; SiCH$_3$), 18.8-18.9 (m, 2C; NCHCH$_3$), D_1 42.9, $D_{2/3}$ 43.0, $D_{2/3}$ 43.1 [6C; N(CH$_3$)$_2$], 64.1-64.5 (2C; NCH), 126.7-127.0, 127.5, 129.6-129.8, 130.8-130.9, 134.4-134.6 (m, 12C; CH$_{ar}$), 134.9, 135.7, 136.0, 136.1 (4C; SiC_{ipso}), 140.2, 140.3, 140.5, 140.6 (2C; NCHC_{ortho}).

{^{1}H}^{29}Si-NMR (119.3 MHz, C_6D_6): δ = D_2 –13.6, D_1 –13.5, –13.3, D_3 –13.1 (2Si).

GC/EI-MS: t_R = 26.38 min [80 °C (1 min) – 30 °C/min-1 – 300 °C (23 min)]; *m*/*z* (%): 212 (43) [($Ph_2SiMeO)^+$], 207 (20) [($PhCHMeNMe_2SiMeO)^+$], 197 (100) [($Ph_2MeSi)^+$], 198 (16), 153 (19), 141 (11).

LC/HR-MS: t_R = 11.09 min [0.3 mL/min H_2O/MeCN (+ 0.1% MeCOOH): 95:5 (2 min) – 80:20 (Δt = 3 min, 3 min) – 0:100 (Δt =12 min, 8 min) – 95:5 (Δt =4 min, 2 min)];

553.30594 *m*/*z* {$C_{34}H_{45}ON_2Si_2$ [M + H]$^+$, (Δ = –1.00676 ppm)}

3.7. Synthesis and crystallization of zinc complex (*S*,*S*,*S*$_{Si}$,*S*$_{Si}$)-6

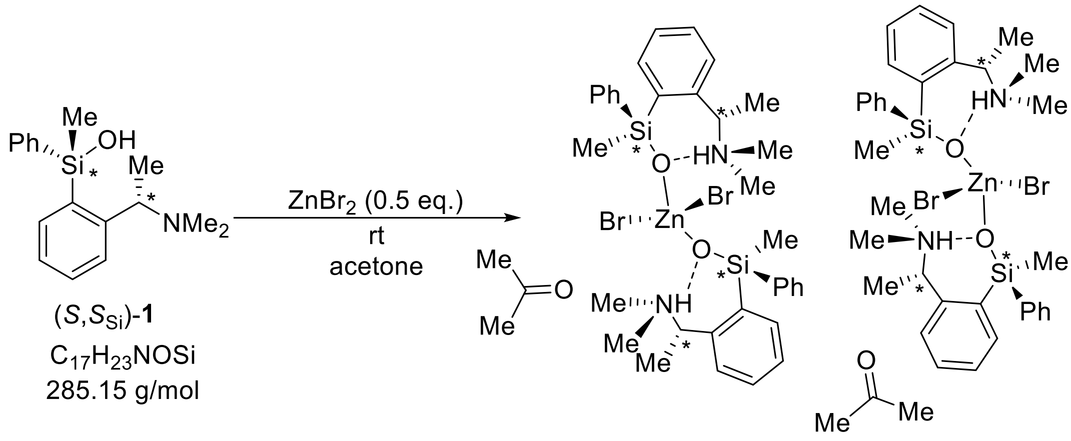

(*S*,*S*$_{Si}$)-**1**
$C_{17}H_{23}NOSi$
285.15 g/mol

(*S*,*S*,*S*$_{Si}$,*S*$_{Si}$)-**6**
2x $C_{34}H_{46}Br_2N_2O_2Si_2Zn \cdot C_3H_6O$
850.12 g/mol

Phenylmethylsilanol (*S*,*S*$_{Si}$)-**1** (0.04 g, 0.15 mmol, 1.0 eq., *d.r.* ≥ 99:1) was dissolved in 1 mL acetone. $ZnBr_2$ (0.02 g, 0.08 mmol, 0.5 eq.) was dissolved in 1 mL acetone and added to the silanol solution. After a few days, the product crystallized from acetone in the form of colorless blocks in space group $P2_12_12_1$ (0.06 g, 0.07 mmol, 97% yield).

Yield: 0.06 g, 0.07 mmol (96.7%, in relation of 2x $C_{34}H_{46}Br_2N_2O_2Si_2Zn{\cdot}C_3H_6O$)

Elemental analysis: Calc.: C: 52.03% H: 6.14% N: 3.28%

Found: C: 52.3% H: 6.2% N: 3.4%

4. Single crystal X-ray diffraction analysis

4.1. Crystal structure of silanol (*S*,*S*$_{Si}$)-1

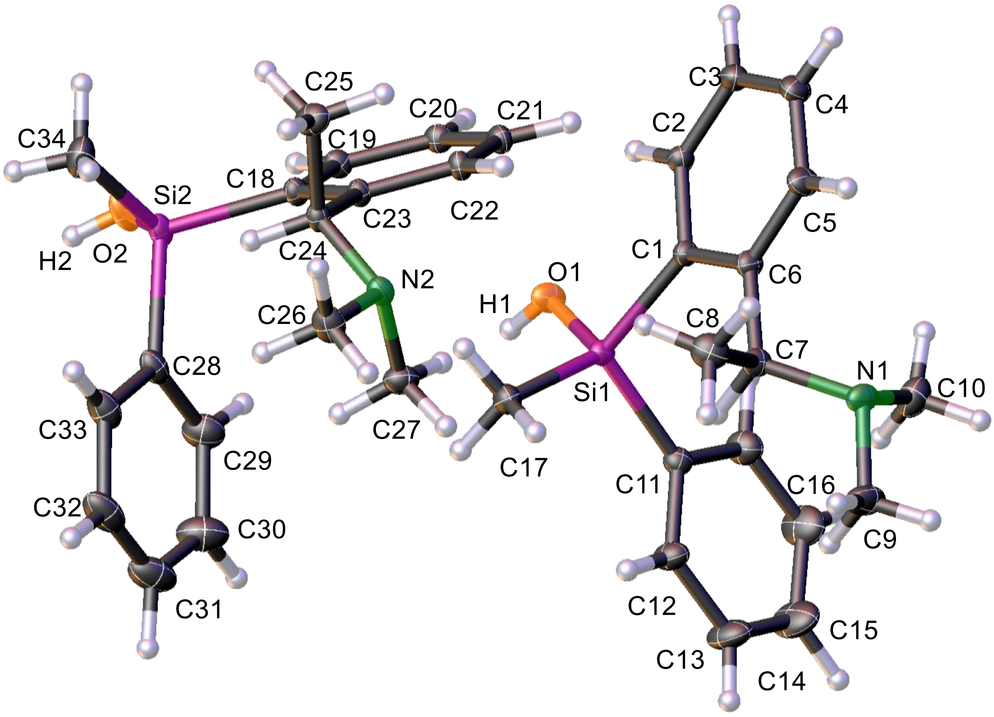

Figure S2: Displacement ellipsoid plot of the molecular structure of silanol (*S*,*S*$_{Si}$)-**1** in the crystal, with ellipsoids drawn at 50% probability. Numbering of hydrogen atoms omitted for clarity. CCDC number: 2166541. (Space group: *P*2$_1$).

Table S1: Crystallographic data and structural refinements of silanol **1.**

**compound**	(S_C,S_{Si})-1
**Empirical formula**	$C_{17}H_{23}NOSi$
**Formula weight [g/mol]**	285.464
**Temperature [K]**	100.0
**Crystal system**	monoclinic
**Space group**	$P2_1$
**Cell dimension [Å]**	8.0345(11)
	11.5847(16)
	17.933(3)
α **[°]**	90
β **[°]**	92.165(2)
γ **[°]**	90
**Volume [Å^3]**	1668.0(4)
**Z**	4
**Calculated density** ***ρ*** **[g/cm^3]**	1.137
**Absorption coefficent** ***μ*** **[mm^{-1}]**	0.137
**F(000)**	616.0
**Crystal size [mm^3]**	0.404 × 0.268 × 0.116
**Radiation**	MoK*α* (*λ* = 0.71073)
**Measuring range 2*θ* [°]**	4.18 – 80.58
**Index ranges**	–14 ≤ h ≤ 14
	–21 ≤ k ≤ 21
	–32 ≤ l ≤ 32
**Reflections collected**	360380
**Independent reflections**	21050 [R_{int} = 0.0515, R_{sigma} = 0.0198]
**Data / restraints / parameters**	21050/1/545
**Goodness-of-fit of F^2**	1.079
**Final *R* indexes [*I*>=2σ (*I*)]**	R_1 = 0.0302, wR_2 = 0.0758
**Final *R* indexes (alle Daten)**	R_1 = 0.0347, wR_2 = 0.0790
**Residual electron density [e Å^{-3}]**	0.36/–0.17
**Flack parameter**	–0.005(10)

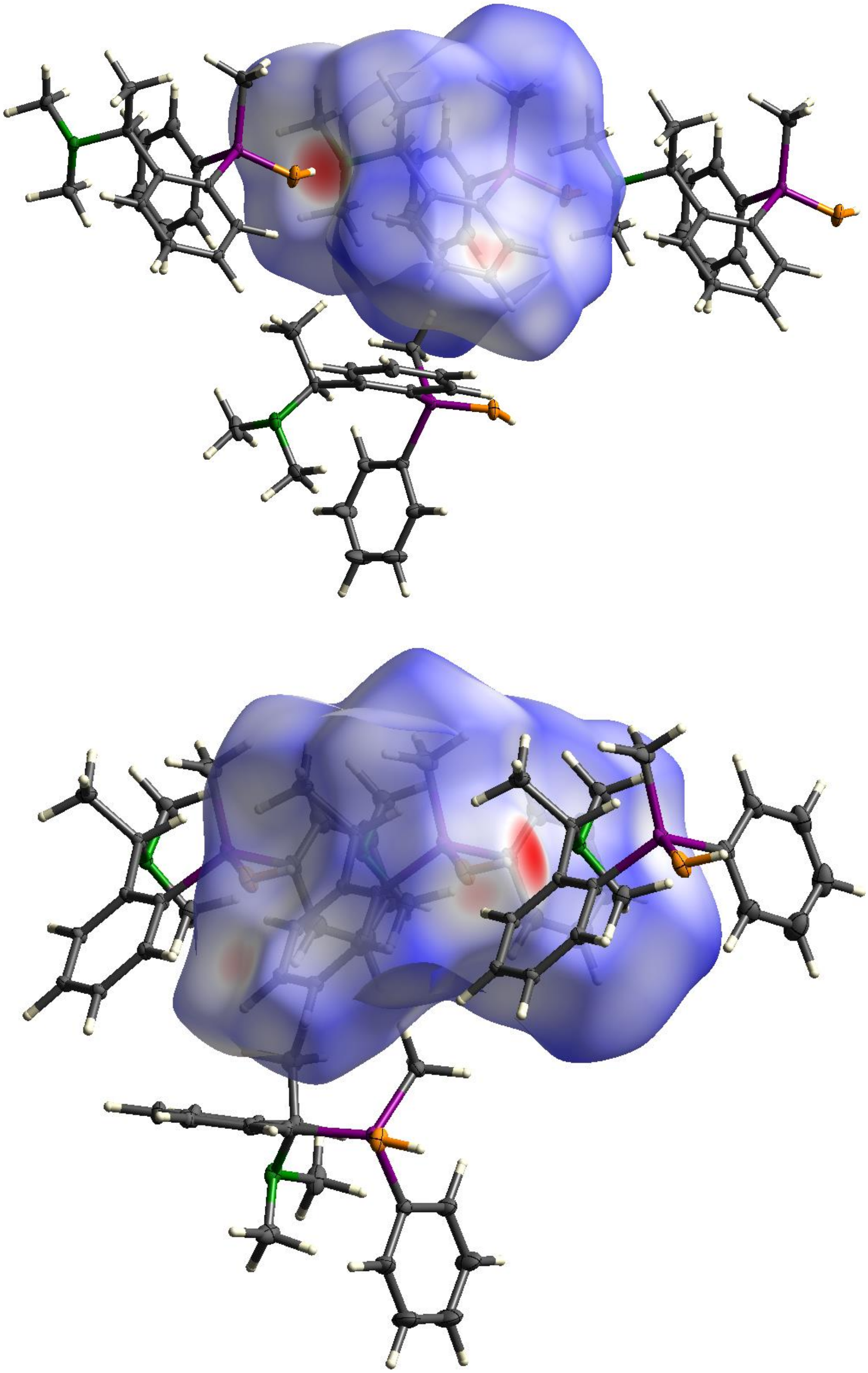

Figure S3: Hirshfeld-surfaces of silanol (*S*,*S*Si)-**1**.

4.2. Crystal structure of zinc complex 6

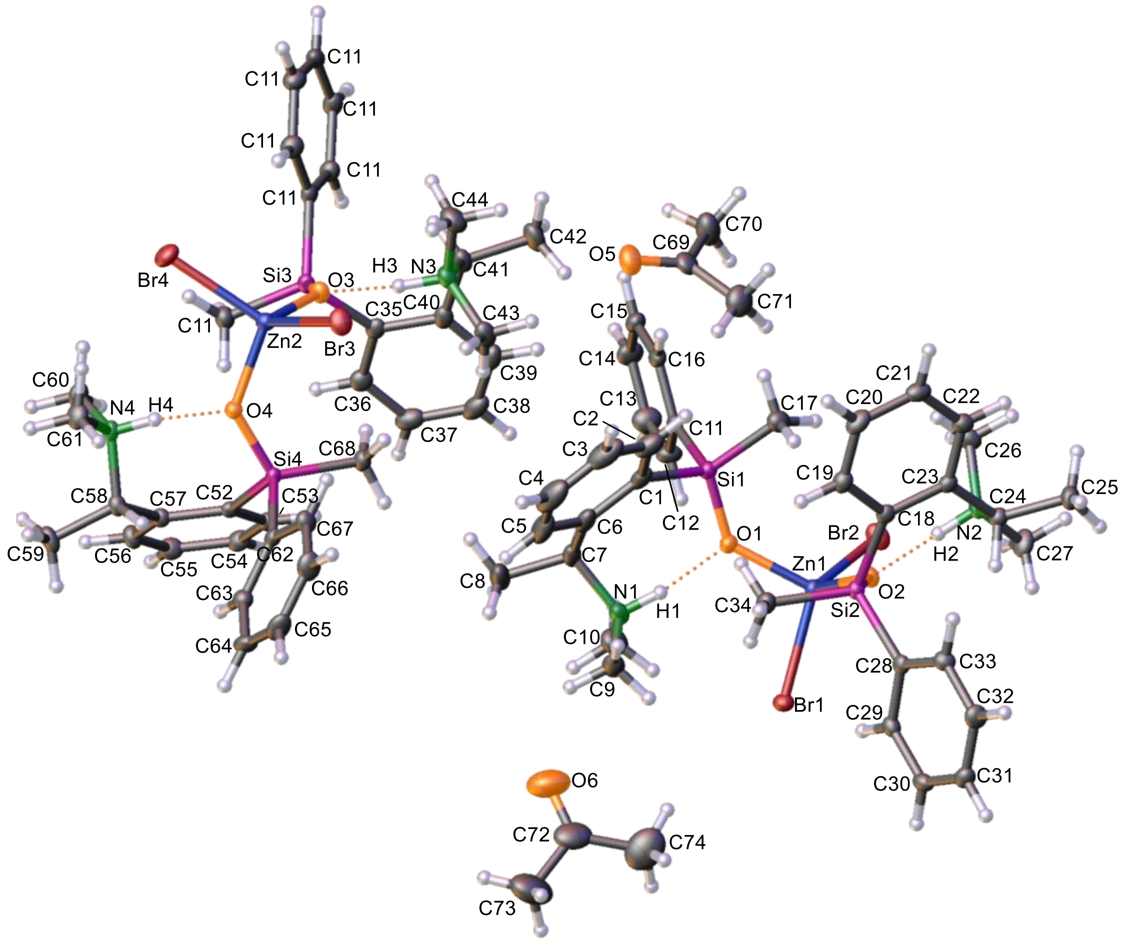

Figure S4: Displacement ellipsoid plot of the molecular structure of zinc complex **6** in the crystal, with ellipsoids drawn at 50% probability. Numbering of hydrogen atoms omitted for clarity. CCDC number: 2166543. (Space group: $P2_12_12_1$).

Table S2: Crystallographic data and structural refinements of zinc complex **6.**

compound	(S,S,S_{Si},S_{Si})-**6**
**Empirical formula**	$C_{37}H_{52}Br_2N_2O_3Si_2Zn$
**Formula weight [g/mol]**	854.17
**Temperature [K]**	100.00
**Crystal system**	orthorhombic
**Space group**	$P2_12_12_1$
**Cell dimension [Å]**	10.9703(5)
	21.0575(10)
	34.8678(14)
α **[°]**	90
β **[°]**	90
γ **[°]**	90
**Volume [Å^3]**	8054.7(6)
**Z**	8
**Calculated density** ***ρ*** **[g/cm^3]**	1.409
**Absorption coefficent** ***μ*** **[mm^{-1}]**	2.689
**F(000)**	3520.0
**Crystal size [mm^3]**	0.166 × 0.158 × 0.14
**Radiation**	MoKα (λ = 0.71073)
**Measuring range 2*θ* [°]**	3.868 – 56.626
**Index ranges**	–14 ≤ h ≤ 14
	–28 ≤ k ≤ 28
	–46 ≤ l ≤ 46
**Reflections collected**	322173
**Independent reflections**	20020 [R_{int} = 0.0448, R_{sigma} = 0.0229]
**Data / restraints / parameters**	20020/0/883
**Goodness-of-fit of F^2**	1.044
**Final *R* indexes [*I*>=2σ (*I*)]**	R_1 = 0.0166, wR_2 = 0.0395
**Final *R* indexes (alle Daten)**	R_1 = 0.0186, wR_2 = 0.0400
**Residual electron density [e Å^{-3}]**	0.23/–0.31
**Flack parameter**	0.0108(11)

The crystals proved to be very difficult to dissolve in the solvents available. Due to the solvent molecules in the solid structure, the attempts to record a powder diffractogram of the crystals in order to confirm the homogeneity of the isolated crystals failed. Instead, the lattice constants were determined from ten randomly selected crystals, which were the same for all crystals.

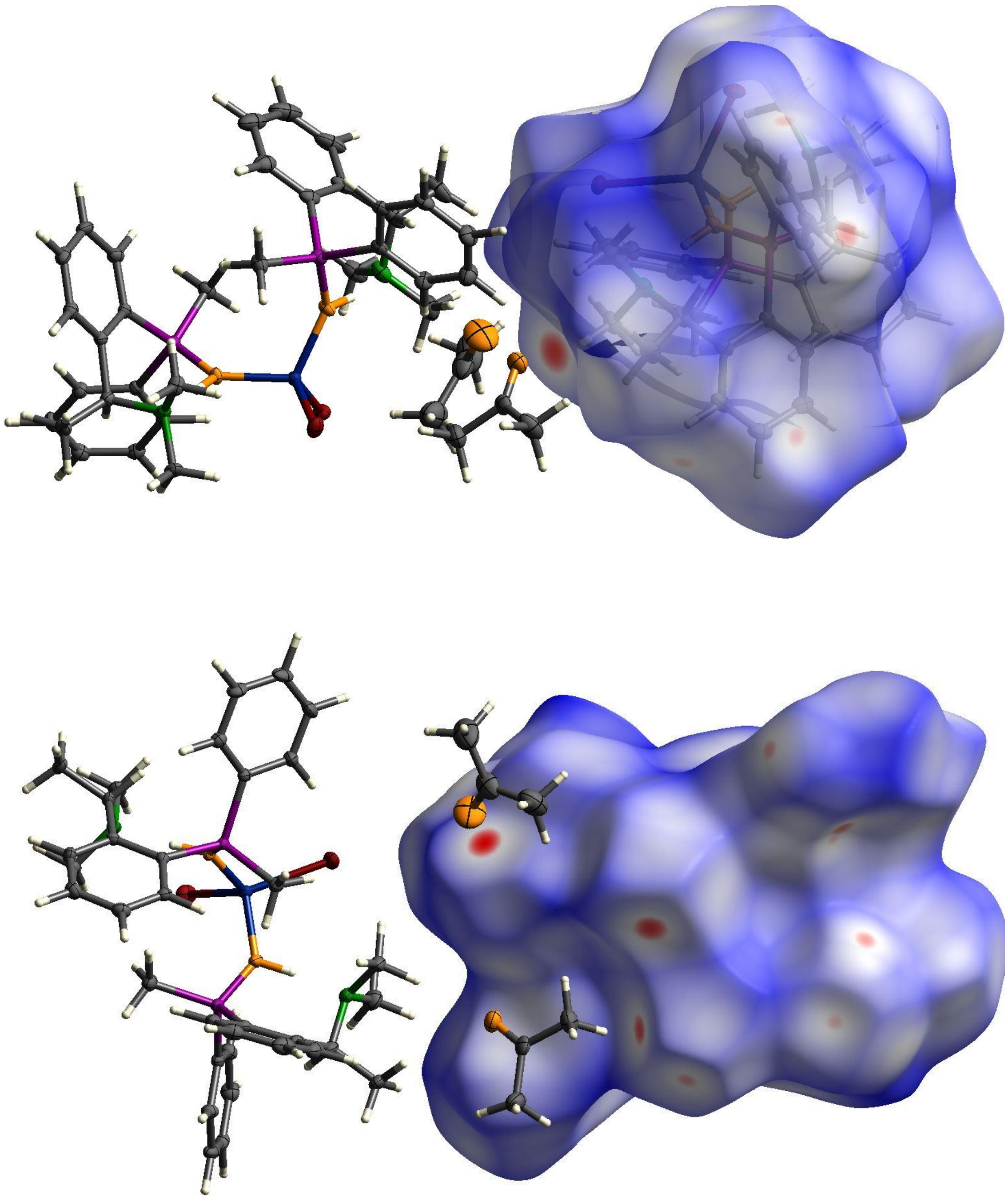

Figure S5: Hirshfeld-surfaces of zinc complex **6**.

4.3. Diastereomeric ratios and absolute configuration

To determine the diastereomeric ratios, the diastereomeric signals of the methyl groups of the amine group of the molecules were used from ^{1}H-NMR spectra.

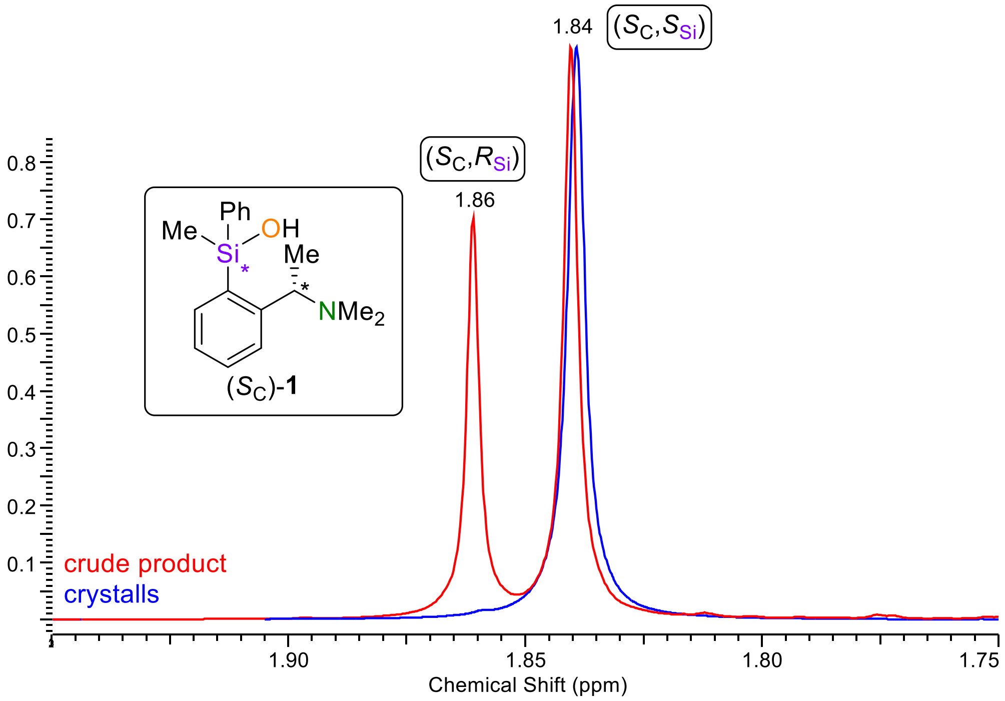

Figure S6: ^{1}H-NMR of silanol **1** (red: crude product, blue: crystals).

The absolute configuration of silanol **1** was determined by X-ray diffraction. By measuring individual crystals and back-determination by NMR spectroscopy, this could be verified for the totality of the crystals formed.

4.4. Verification of diastereomeric ratios

The diastereomeric ratios of silanol were verified by adding a shift reagent to a mixture of the silanol from the four diastereomers [(S,S_{Si}), (S,R_{Si}), (R,S_{Si}), (R,R_{Si}) and (S,S_{Si})] and to the stereochemical pure compound (S,S_{Si}). Subsequently, the ^{1}H- and ^{29}Si-NMR spectra of the compounds were recorded.

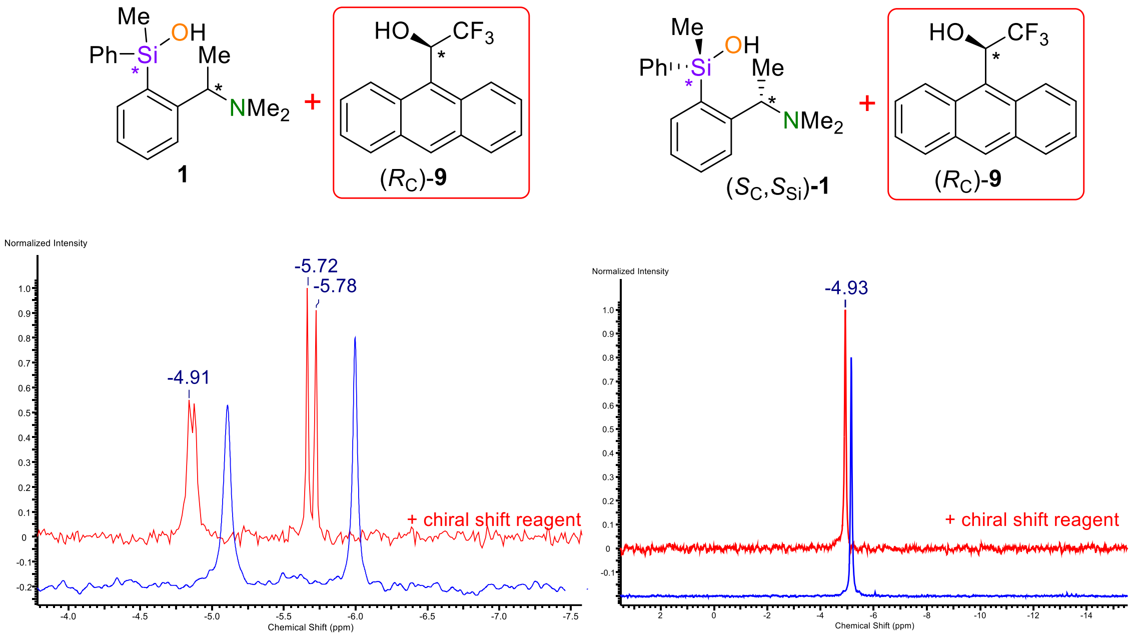

Figure S7: ^{29}Si-NMR spectra of silanol **1** without (blue) and with chiral shift reagent **9** (red) for a mixture of isomers of **1** and for the diastereomeric pure crystals of **1**.

5. NMR studies with silanol 1

5.1. Silanol 1 in benzene-d_6

The silanol (S,S_{Si})-**1** (34.4 mg, *d.r.* = 97:3) was dissolved in benzene-d_6 (0.6 mL) and ^{1}H- and ^{29}Si-NMR spectra were measured over a period of 172 h.

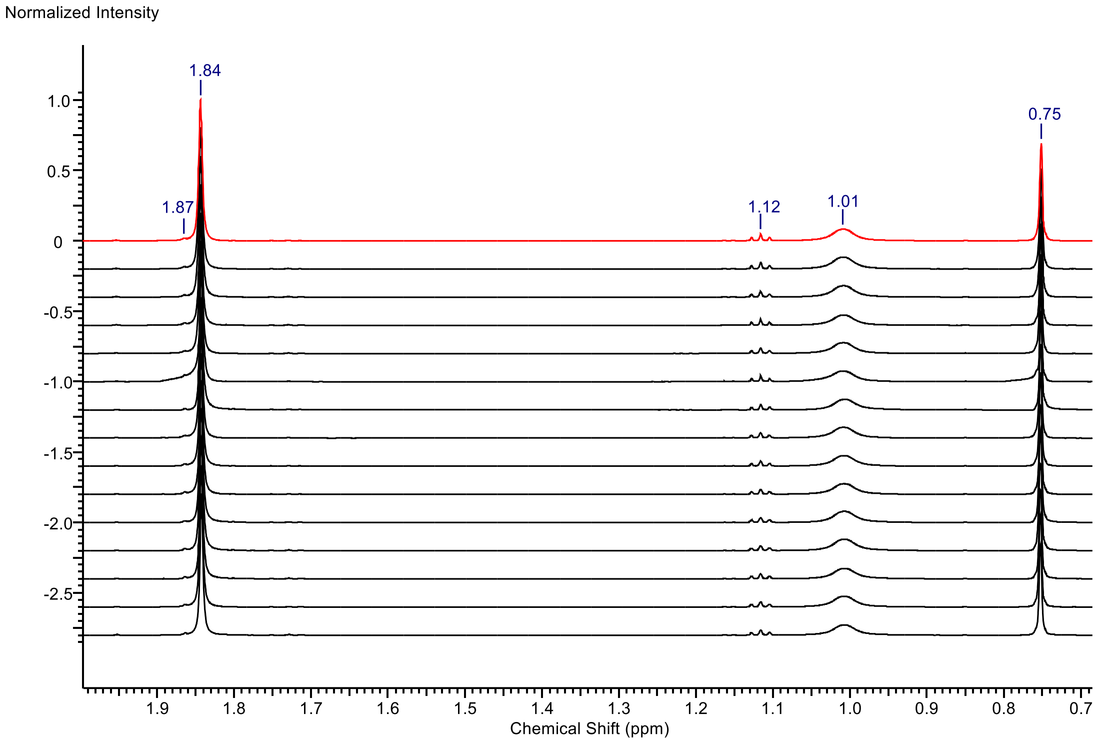

Figure S7: ^{1}H-NMR spectra section (600 MHz) of silanol **1** in C_6D_6 over 172 h (15 spectra, each spectrum recorded after additional 12 h) (Offset Y = 0.2).

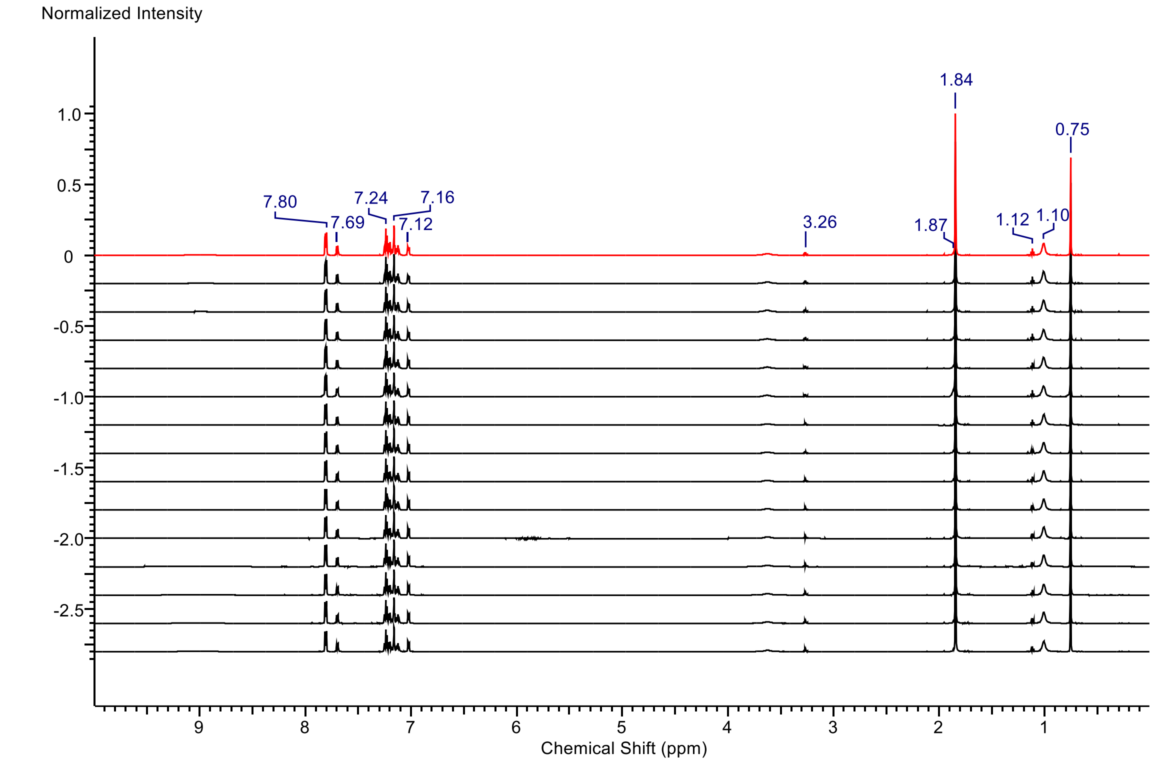

Figure S8: ^{1}H-NMR spectra (600 MHz) of silanol **1** in C_6D_6 over 172 h (15 spectra, each spectrum recorded after additional 12 h) (Offset Y = 0.2).

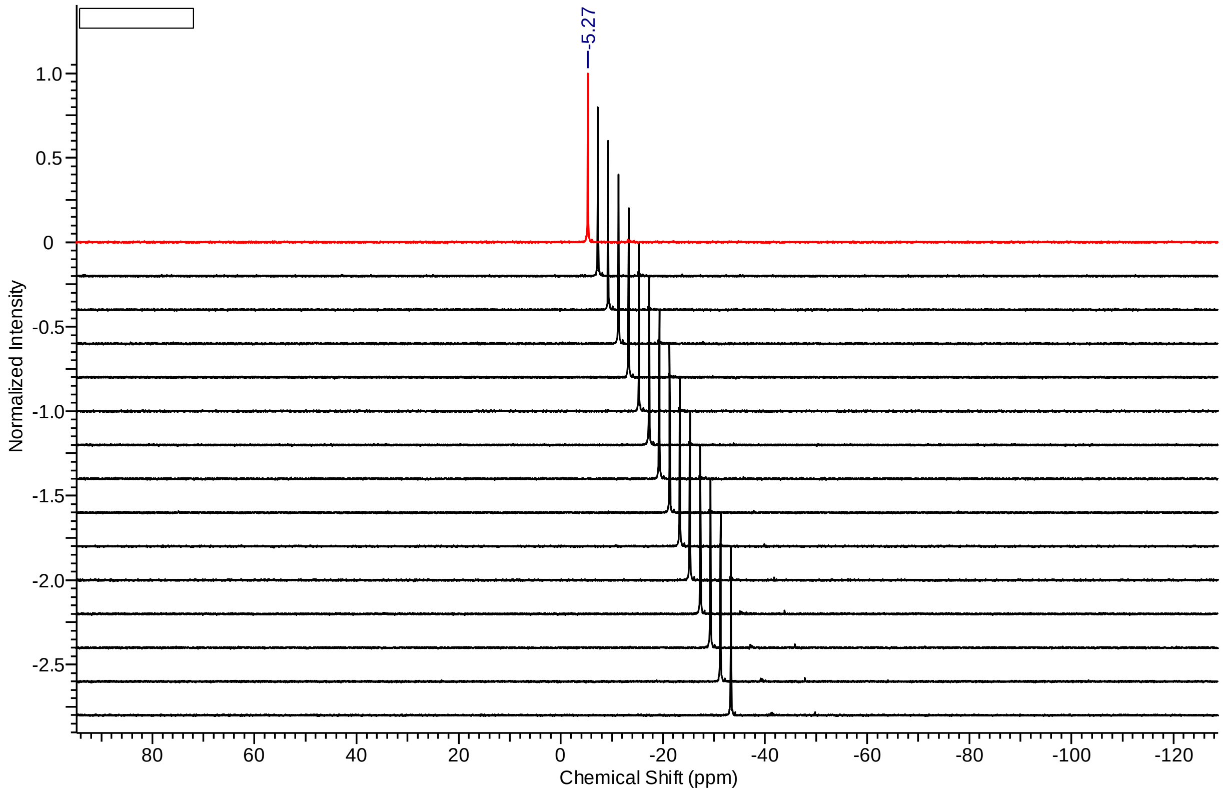

Figure S9: ^{29}Si-NMR spectra (119 MHz) of silanol **1** in C_6D_6 over 172 h (15 spectra, each spectrum recorded after additional 12 h), (Offset: X: 2, Y: 0.2) .

5.2. Silanol 1 in Et_2O

The silanol (S,S_{Si})-**1** (22.7 mg, *d.r.* ≥ 99:1) was dissolved in Et_2O (0.6 mL). ^{1}H- and ^{29}Si-NMR spectra were measured over a period of 4 days.

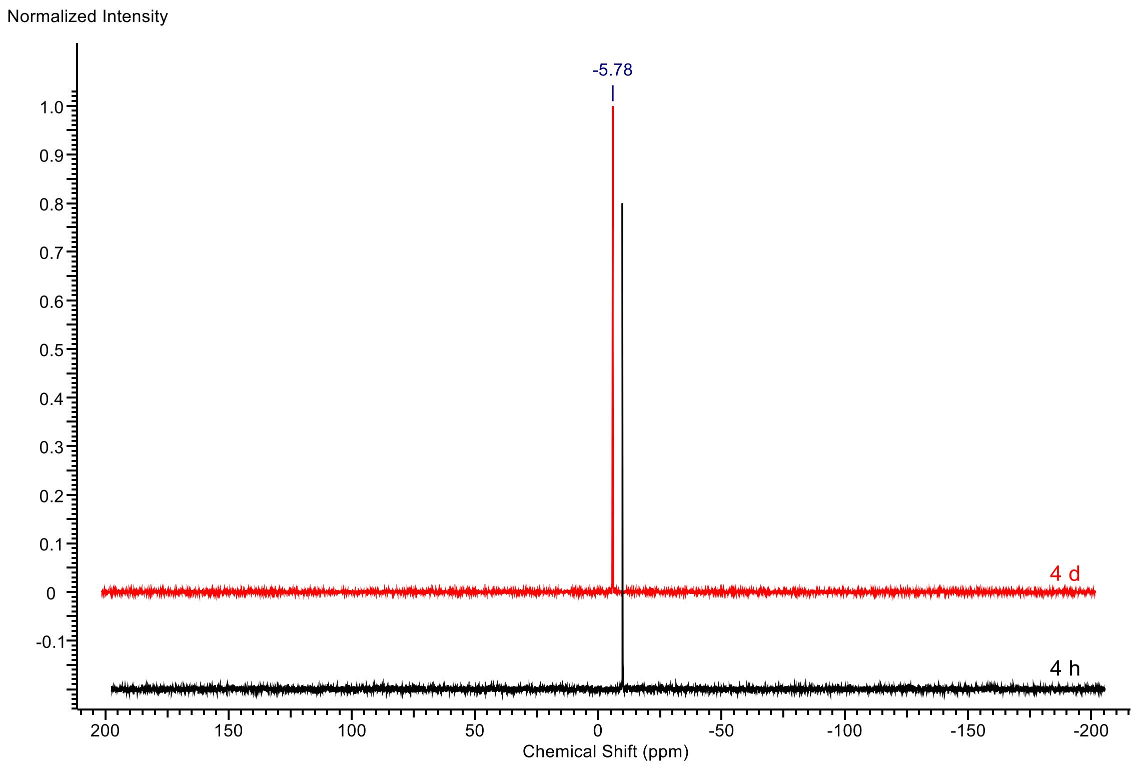

Figure S10: ^{29}Si-NMR spectra from silanol **1** in Et_2O (C_6D_6 internal standard) over 4 days (black: after 4 h, red: after 4 days).

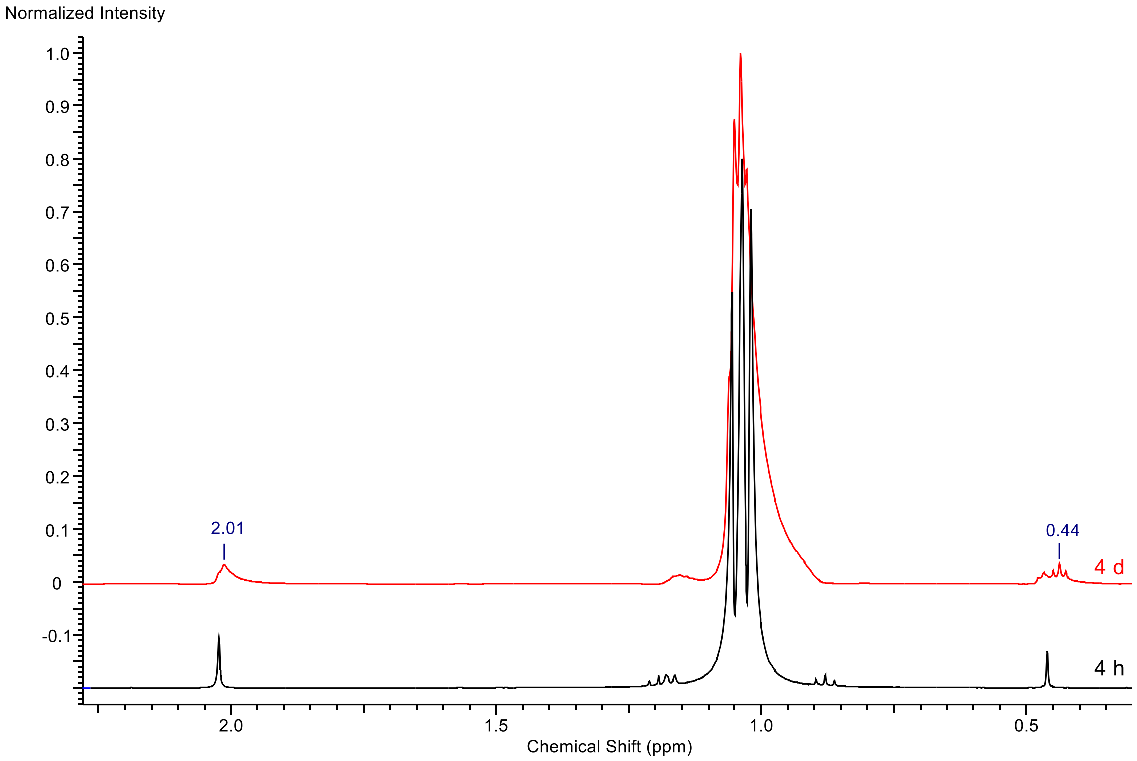

Figure S11: ^{1}H-NMR spectra from silanol **1** in Et_2O over 4 days (blue: 4 h, red: 4 days).

5.3. Silanol 1 in THF

The silanol (S,S_{Si})-**1** (22.3 mg, *d.r.* = 98:2) was dissolved in THF (0.6 mL). A benzene-d_6 capillary was added to the NMR-tube and ^{1}H- and ^{29}Si-NMR (inversed gated) spectra were measured over a period of 7 days.

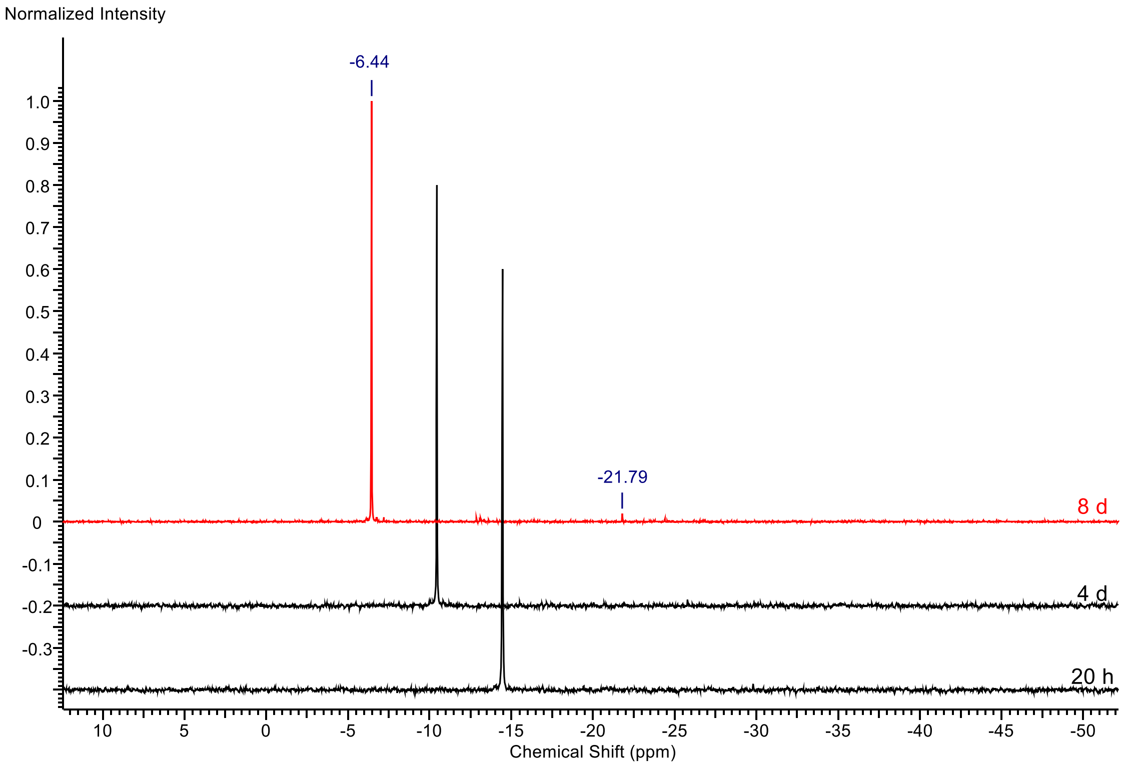

Figure S12: ^{29}Si-NMR spectra from silanol **1** in THF (C_6D_6 internal standard) over 8 days (blue: 20 h, green: 4 days, red: 8 days).

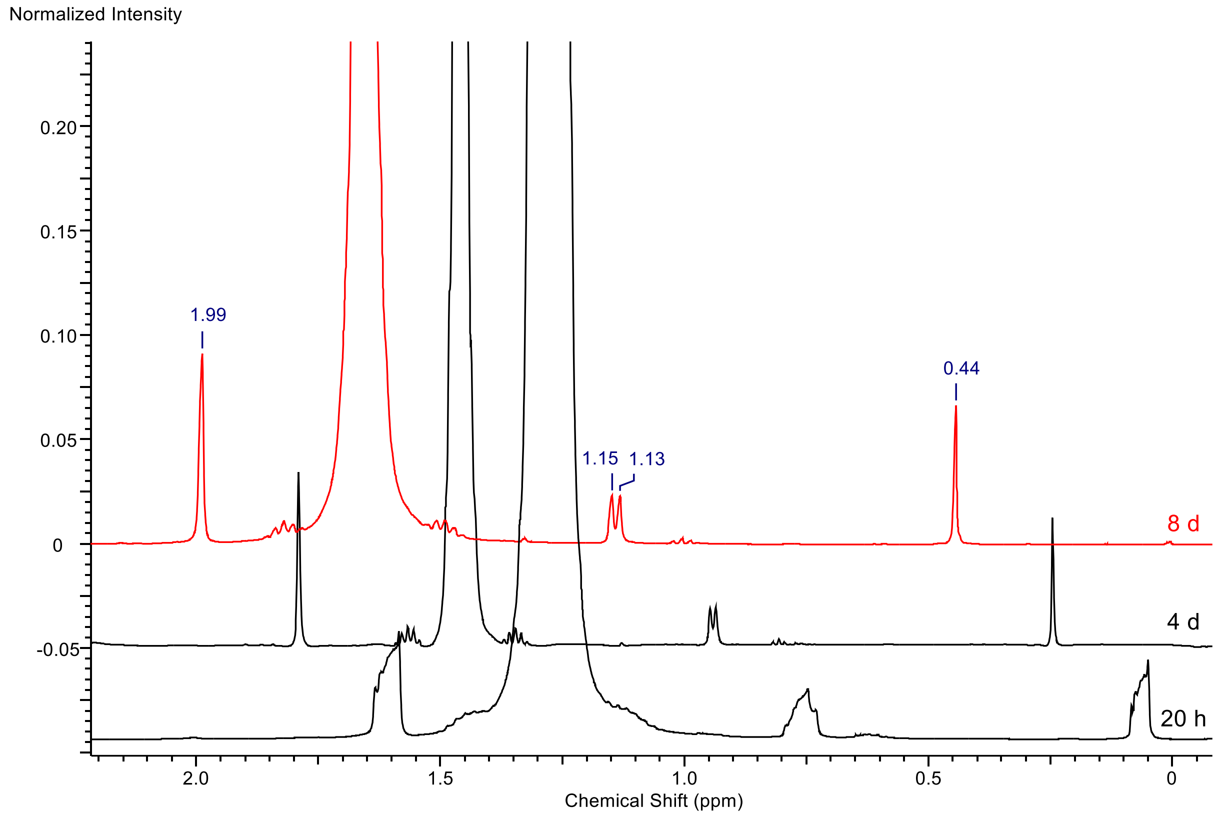

Figure S13: ^{1}H-NMR Spectra from silanol **1** in THF (C_6D_6 internal standard) over 8 days blue: 20 h, green: 4 days, red: 8 days.

6. NMR spectra of compounds

6.1. NMR spectra of amine 2

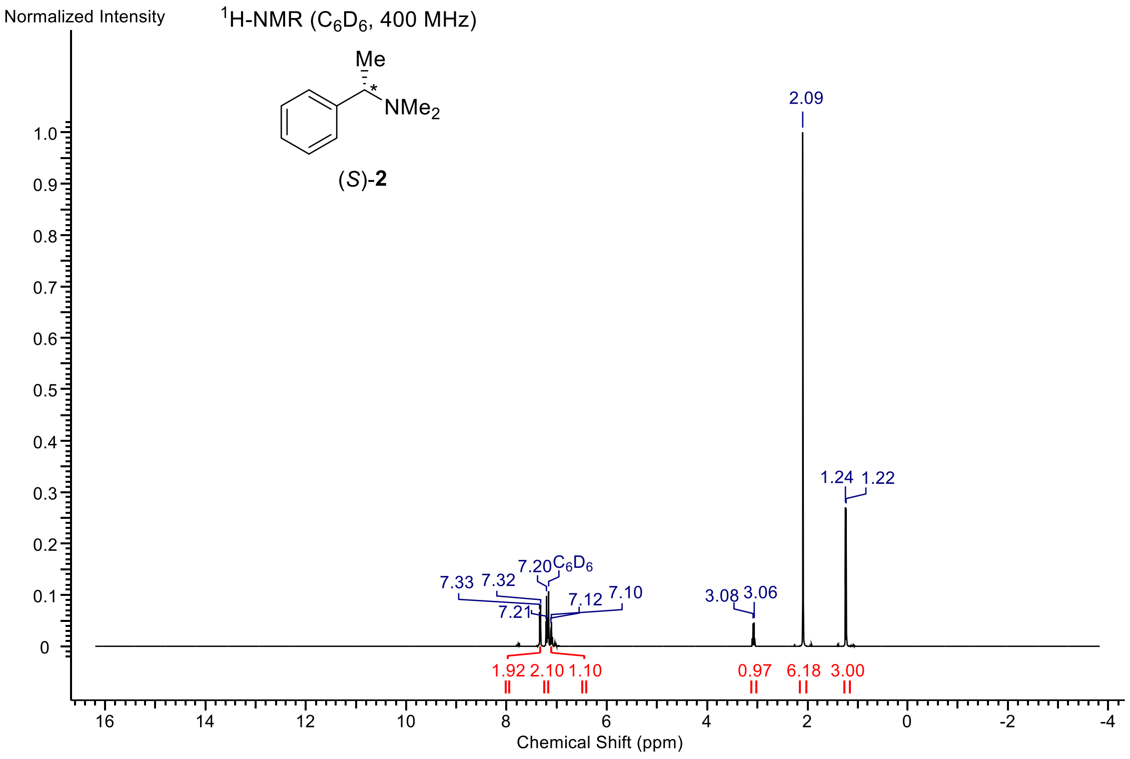

Figure S14: ^{1}H-NMR spectrum of amine (*S*)-**2** in C_6D_6 (400 MHz).

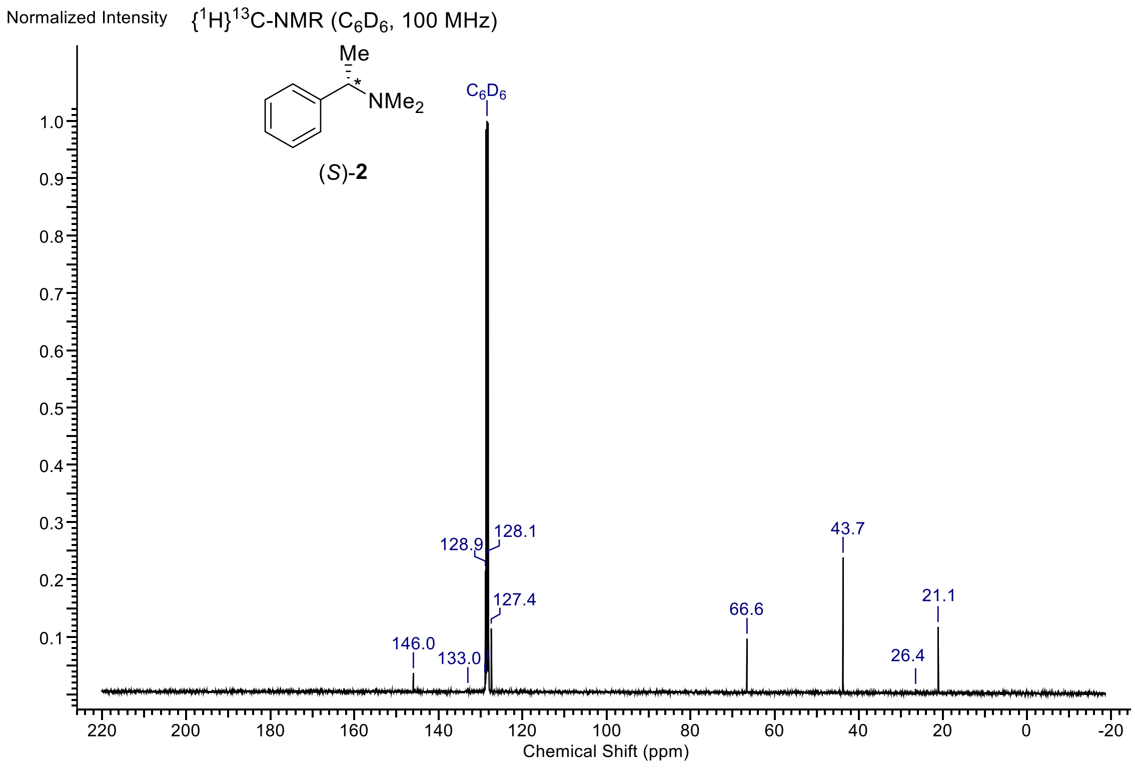

Figure S15: {^{1}H}^{13}C-NMR spectrum of amine (*S*)-**2** in C_6D_6 (100 MHz).

6.2. NMR spectra of methoxysilane 3

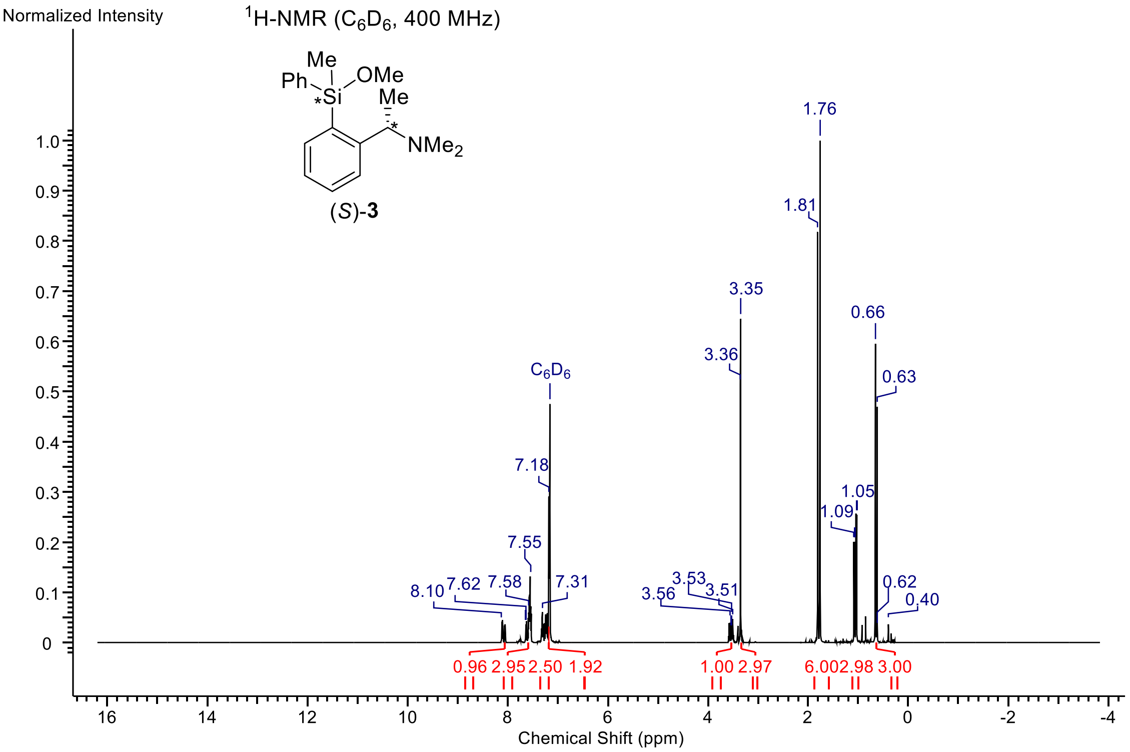

Figure S16: ^{1}H-NMR spectrum of methoxysilane (*S*)-**3** in C_6D_6 (400 MHz).

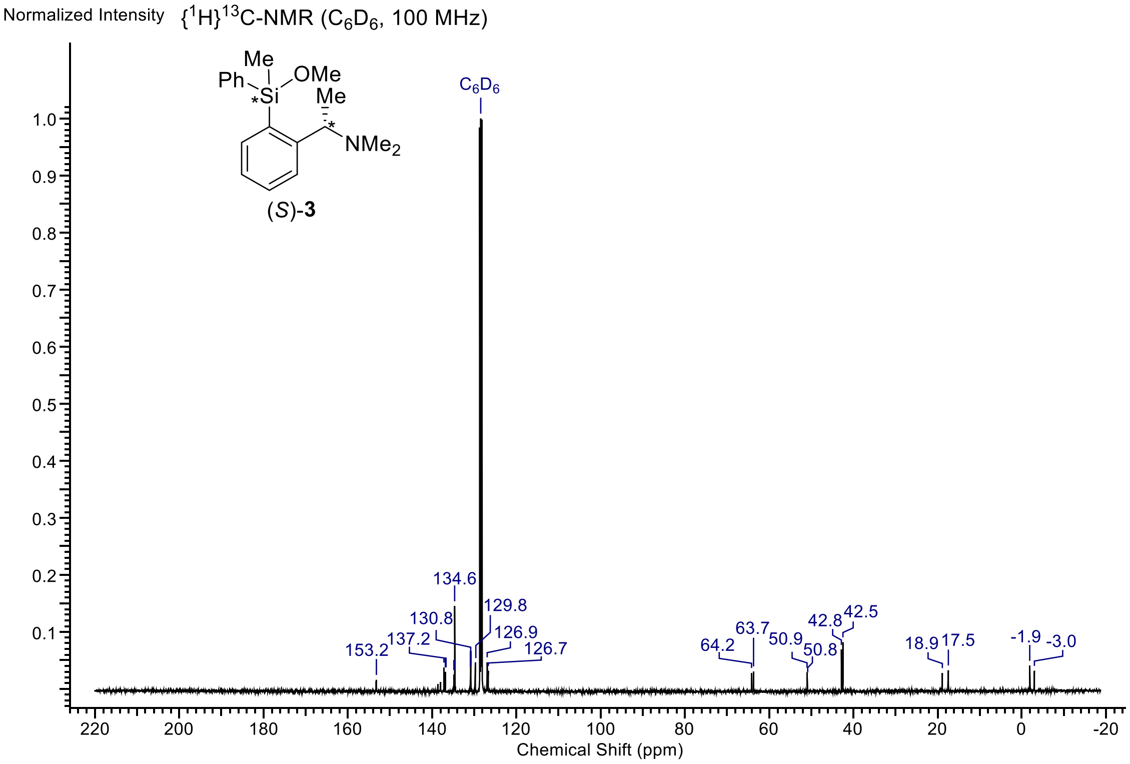

Figure S17: {^{1}H}^{13}C-NMR spectrum of methoxysilane (*S*)-**3** in C_6D_6 (100 MHz).

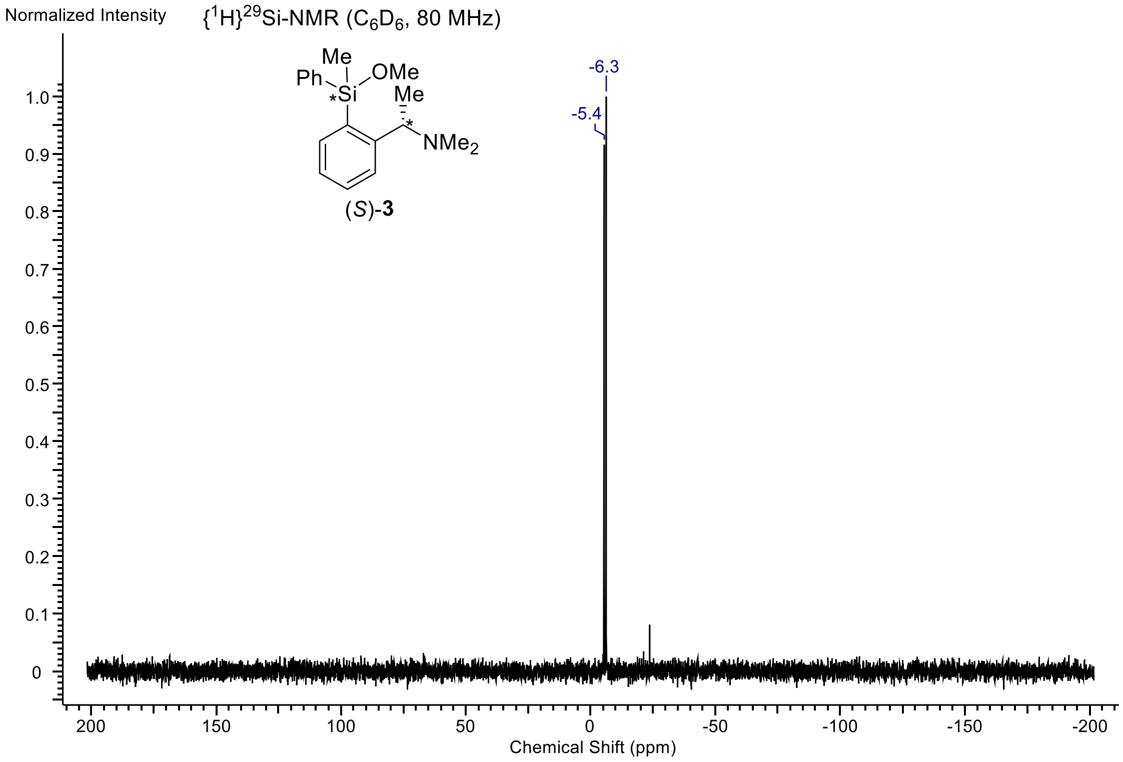

Figure S18: {^{1}H}^{29}Si-NMR spectrum of methoxysilane (*S*)-**3** in C_6D_6 (80 MHz).

6.3. NMR spectra of silanol (S,S_{Si})-1

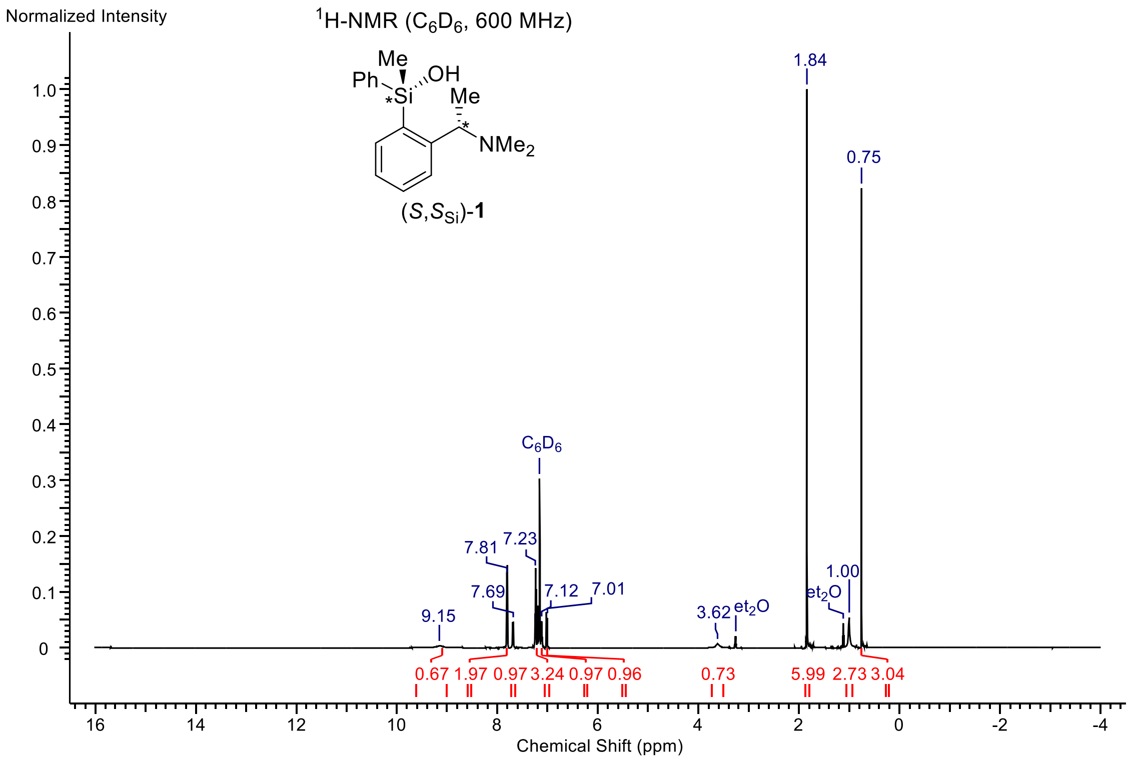

Figure S19: ^{1}H-NMR spectrum of silanol (S,S_{Si})-**1** in C_6D_6 (600 MHz).

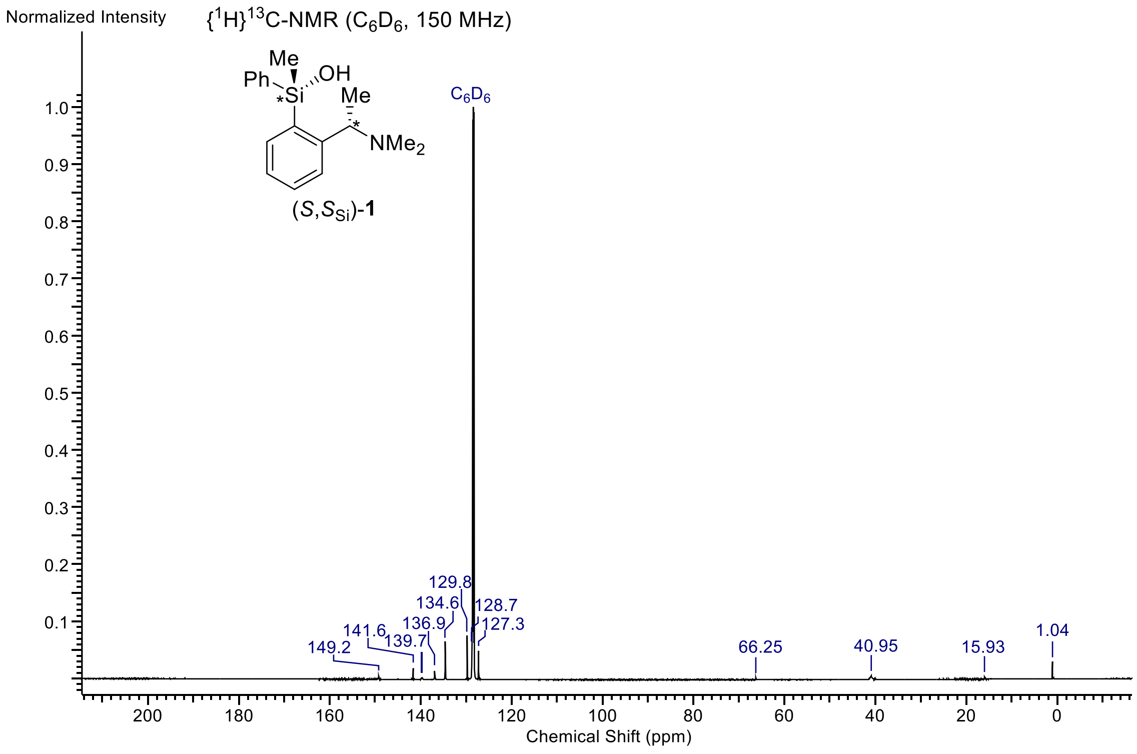

Figure S20: {^{1}H}^{13}C-NMR spectrum of silanol (S,S_{Si})-**1** in C_6D_6 (150 MHz).

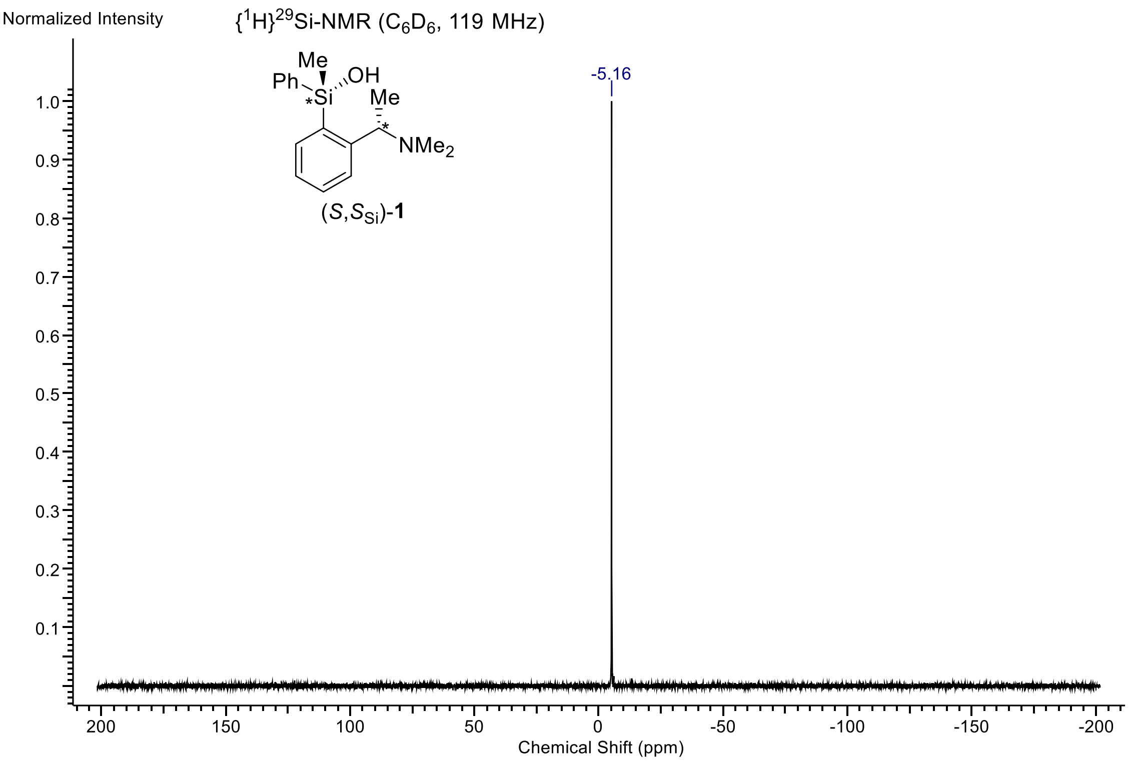

Figure S21: {^{1}H}^{29}Si-NMR spectrum of silanol (*S*,*S*$_{Si}$)-**1** in C_6D_6 (119 MHz).

6.4. NMR Spectra of siloxane 5

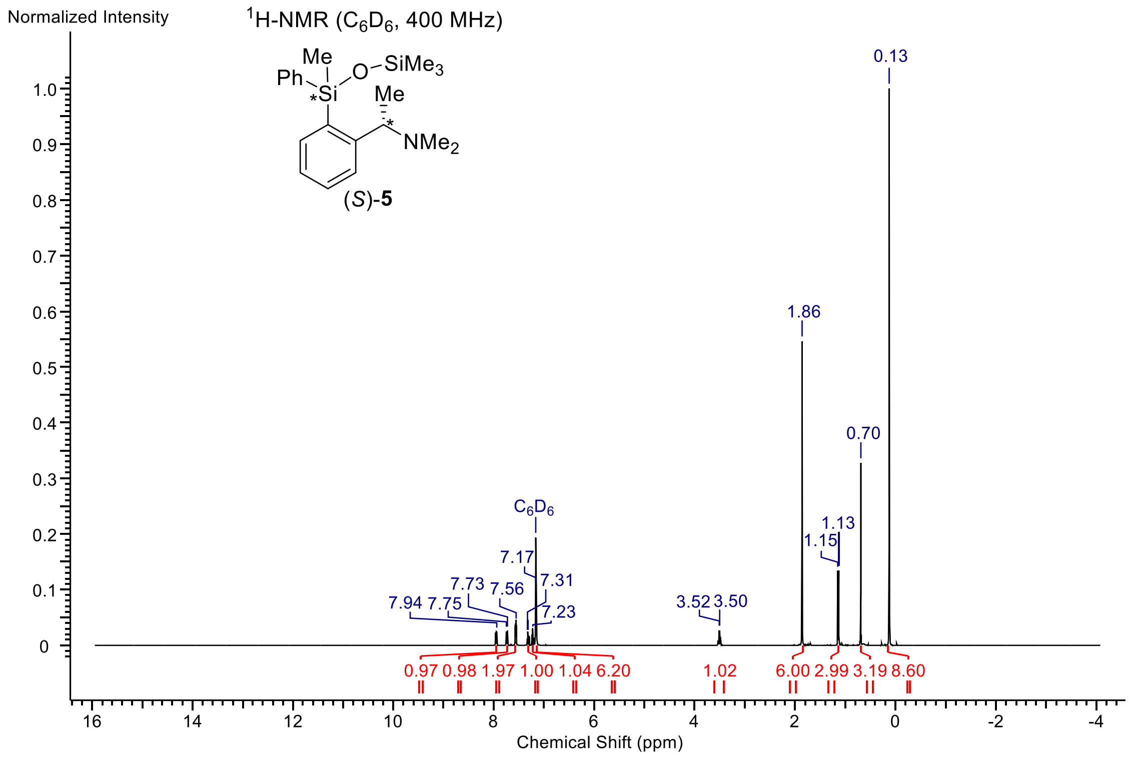

Figure S22: ^{1}H-NMR spectrum of siloxane (*S*)-**5** in C_6D_6 (400 MHz).

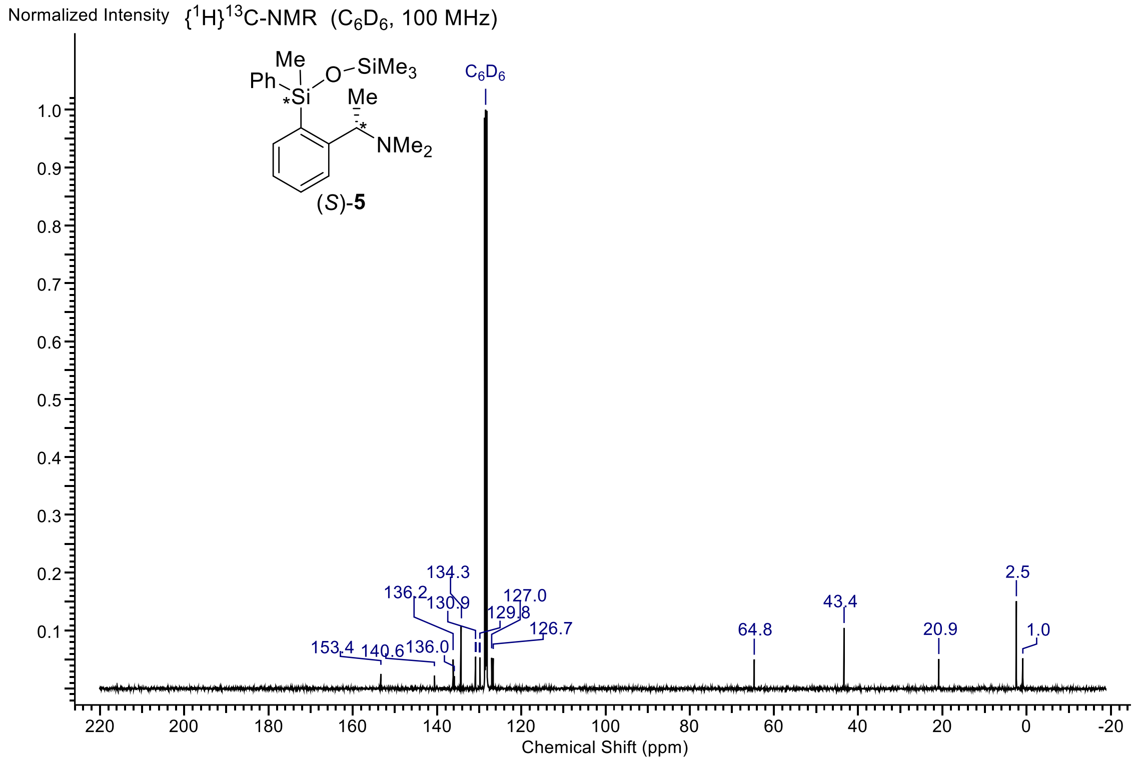

Figure S23: {^{1}H}^{13}C-NMR spectrum of siloxane (*S*)-**5** in C_6D_6 (100 MHz).

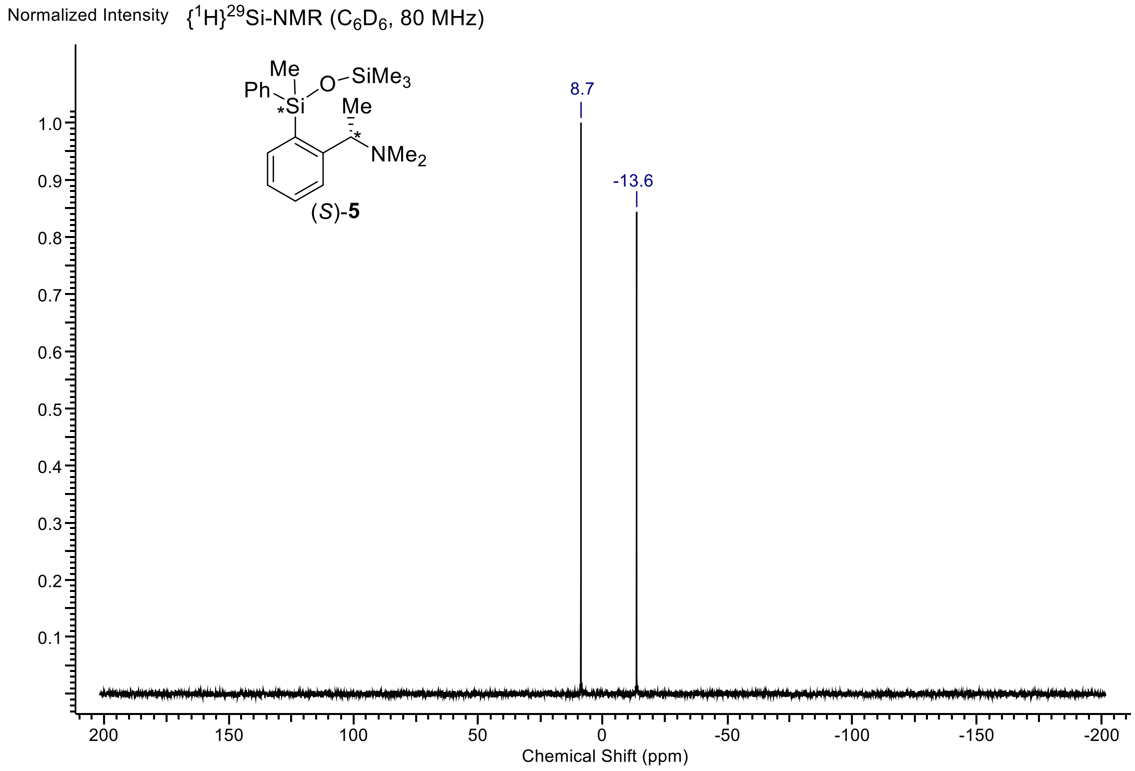

Figure S24: {^{1}H}^{29}Si-NMR spectrum of siloxane (*S*)-**5** in C_6D_6 (80 MHz).

7. Mass spectrometric studies

7.1. Experiment with H_2O-^{18}O

Procedure:

One solution of silanol (S,S_{Si})-**1** ($c_{silanol}$ = 10 mg/mL, *d.r.* = 97:3) was prepared in in THF(HPLC grade), respectively. Subsequently, ^{18}O-labeled water (97% ^{18}O content, c_{water} = 30 µL/mL) was added to the respective solution. The solution was stored at 4 °C and samples were taken from the solution at defined time intervals. The addition of the water marks the starting point (t = 0 h) of the experiment.

For the sample collection, 2 µL of the solution was taken and mixed with an excess of hexamethyldisilazane (20 µL). The sample was heated for 15 min at 50 °C in an ultrasonic bath. After dilution with 10 mL THF $MgSO_4$ (2 g) was added to dry the sample and, after vertexing three times, the solution was centrifuged and taken for the GC/EI-MS measurement. All samples were determined in triplicate and the mean values of the corresponding m/z values were determined to reduce errors in the determinations.

The diastereomer ratio of the silanol diastereomers results from the ratio of the signal areas of the peaks to each other multiplied by the correction factors of the calibration. From the ratio of the detected values for the 244 and 246 m/z values, the enrichment of ^{18}O in the respective silanol diastereomers was determined.

GC/EI-MS parameters:

The sample was injected at 40 °C (*splitless-mode;* PTV).

Temperature program:

[80 °C (0.5 min) – 40 °C/min – 180 °C (25 min) – 40 °C/min – 280 °C (2 min)]

The gas flow was constant 0.7 mL/min. After chromatographic separation, the gas was transported via the 280 °C warm transfer line into the 300 °C hot ion source. Detection was performed in the m/z range of 200-600 in positive mode at 8-28 min.

Note: An unknown component was observed in some of the measured spectra (313 m/z). However, the measurement of the silylation reagent used (hexamethyldisilazane) showed that this is an impurity of the reagent and not a side compound of the experimental series.

7.2. Calibration:

For the calibration, solutions of silanol **1** [*d.r.* (*S*,S_{Si}/R_{Si}) = 57:43] with different concentrations were prepared and the response signal was measured during GC/EI-MS measurements. This results in the correction factor for determining the actual concentrations and diastereomeric ratios of silanol **1**.

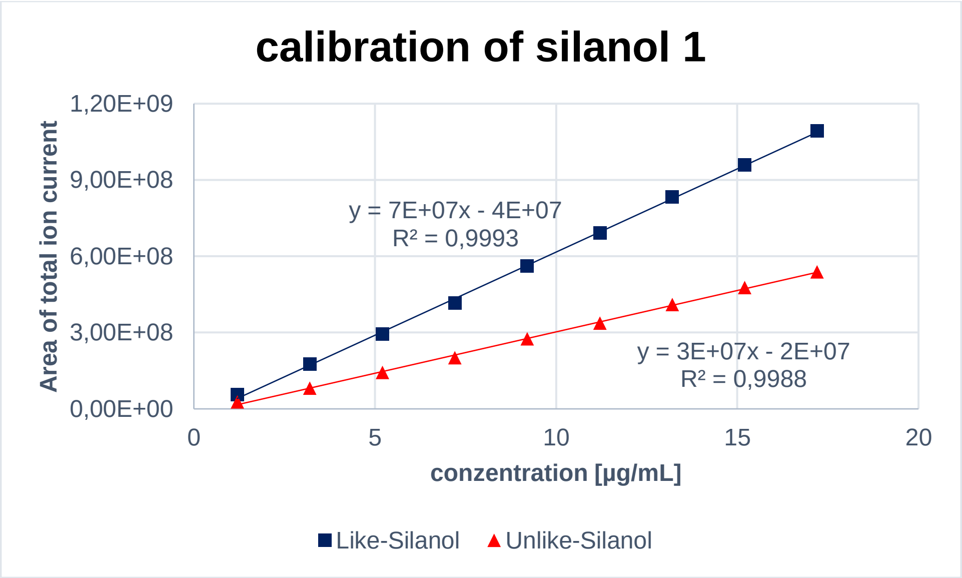

Figure S25: Calibration of silanol **1** in GC/EI-MS by measurement of derivate siloxane **5** in different concentrations; *Like*-silanol ≙ (*S*,S_{Si})-**1**, *Unlike*-silanol ≙ (*S*,R_{Si})-**1.**

7.3. Additional experiment:

In addition to the experiment in THF (4 °C) a similar experiment was carried out at room temperature (rt). The diastereomeric ratio of (S_{Si}/R_{Si}) doesn't show significant changes during the experiment. The proportions of ^{18}O in both isomers of silanol **1** increase during the experimental period.

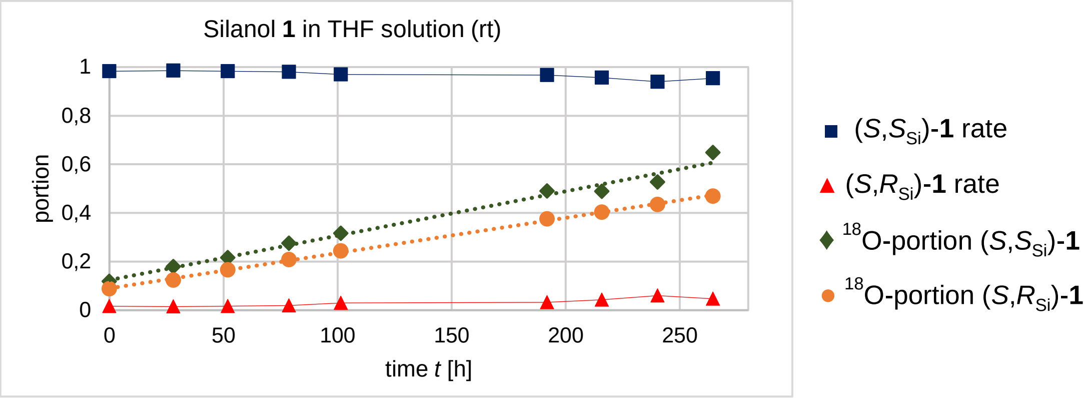

Figure S26: Exchange reactions of silanol **1** in THF at room temperature. Conditions: $c_{[silanol]}$ = 10 mg/mL, $c[^{18}OH_2]$ = 30 µL/mL; T = rt.

7.4. Values of the GC/EI-MS experiment: silanol 1 in THF

Table S3: Data of the measurements of the GC/EI-MS experiment in THF 4 °C. Diastereomer 1 ≙ derivate of (*S*,*R*$_{Si}$)-**1**; Diastereomer 2 ≙ derivate of (*S*,*S*$_{Si}$)-**1**.

**Measurement**	**time** *t* **[h]**	**m/z 264**		**m/z 266**	
		Area Diastereomer 1	Area Diastereomer 2	Area Diastereomer 1	Area Diastereomer 2
1THF1	0	788379	41388290	85221	4030941
1THF2		730635	41644688	87331	3951385
1THF3		787495	45497330	107995	4447786
2THF1	24	698882	38694021	92585	4513780
2THF2		862191	45889876	103728	5290159
2THF3		797530	37957083	100222	4413215
3THF1	48	1063066	62488969	135704	8434718
3THF2		1269125	73229096	164010	9927096
3THF3		1334651	74526000	160647	10245622
4THF1	72	1184179	58408466	176147	9261760
4THF2		1094242	52269434	164273	8319456
4THF3		904249	48533710	129245	7679721
5THF1	120	1025631	53293400	182402	11722541
5THF2		984202	56216303	152029	12422653
5THF3		1090643	62388989	189441	13560907
6THF1	148	922000	53267233	156914	13359445
6THF2		915290	56637005	161814	14121419
6THF3		785360	51009474	138853	12891848
7THF1	172	1171671	66968075	242543	18955370
7THF2		858451	49658289	163268	14281331
7THF3		1063722	66285111	201890	19055337
8THF1	196	1041558	54875222	210311	17756480
8THF2		973083	57258017	222467	18622427
8THF3		925300	55030663	203614	17958875
8THF4		1000838	51136689	198255	16562341

8THF5		919948	53160518	185329	17385957
8THF6		890009	54609688	184099	17784715
9THF1	220	792567	37975747	172765	13635863
9THF2		776457	38621254	188509	13790346
9THF3		892106	43783523	178170	15761280
10THF1	244	884037	47460923	198272	19023982
10THF2		896973	49194806	220499	20196620
10THF3		855378	45231930	193468	18200258
11THF1	317	1001127	56173964	268680	31335522
11THF2		951371	53152986	263417	28944063
11THF3		898803	50888489	260308	28544213
12THF1	340	673020	38911881	292273	23483397
12THF2		707705	39917764	284657	24039510
12THF3		646818	34208990	253259	20326352
13THF1	364	330514	17594048	157383	11605431
13THF2		466279	23392023	194138	15344699
13THF3		542368	22903470	205407	14847348
14THF1	388	555536	25736287	211276	18074087
14THF2		523482	27906264	223811	19933067
14THF3		604531	28102467	242324	20032816
15THF1	412	493439	20963636	249868	16178298
15THF2		600914	12926840	241193	18427558
15THF3		535018	22601798	208931	17236666
16THF1	437	738632	33529806	354203	31575765
16THF2		853348	34489864	376863	32759362
16THF3		834451	36557310	427775	34808016
17THF1	483	518566	16190523	224741	17149373
17THF2		536032	17976160	225360	19080056
17THF3		635654	20731078	258472	22281847
18THF1		554449	17906851	236904	19897557

18THF2	508	602322	19266595	224101	21355215
18THF3		615115	19728117	236057	22394919
19THF1	605	552209	16414035	262233	23497800
19THF2		658033	21490828	312928	30171400
19THF3		608030	18483983	265958	25923580
20THF1	821	712963	15199888	424967	33196459
20THF2		681834	13984862	381320	30910818
20THF3		810471	17767988	487744	39371639

Table S4: Data of the measurements of the GC/EI-MS experiment in THF at room temperature. Diastereomer 1 ≙ derivate of (*S*,R_{Si})-**1**; Diastereomer 2 ≙ derivate of (*S*,S_{Si})-**1**.

**Measurement**	**time** *t* **[h]**	**m/z 264**		**m/z 266**	
		Area Diastereomer 1	Area Diastereomer 2	Area Diastereomer 1	Area Diastereomer 2
1THF1	0	1567468	136011378	240685	13390388
1THF2		1456269	136310178	174006	12985307
1THF3		1516915	138573497	196482	13346914
2THF1	28	1581498	184595665	332160	23283913
2THF2		1695384	169600375	374374	25349051
2THF3		1556242	166461231	349555	24806371
3THF1	52	1803361	160668850	457250	31820529
3THF2		1844330	172125044	540657	34265831
3THF3		1826914	179050749	505037	35851695
4THF1	79	1596092	128560228	576411	33638642
4THF2		1569164	136510857	611238	36018707
4THF3		1616300	136741633	623640	36023917
5THF1	102	2807231	149882489	1273539	47974419
5THF2		2862005	157062244	1403999	51432763
5THF3		2808332	158656128	1232194	50390805

6THF1	192	2100346	116995629	2037050	69932931
6THF2		2019854	109573720	1909989	65521812
6THF3		1807667	99877173	1738779	60291267
7THF1	216	~~1244850~~	~~6913494~~	~~257647~~	~~3237191~~
7THF2		2554822	110835721	2343585	74696097
7THF3		2417369	112401422	2408108	75972687
8THF1	240	6768128	142278125	5055148	103646284
8THF2		4398243	136714064	4837478	104807593
8THF3		4365506	139219738	4897680	107257216
9THF1	265	1235275	46036055	1879495	40224947
9THF2		1985117	88450423	3423589	77903817
9THF3		1992758	107288108	3901094	95089123

8. IR studies

8.1. IR spectrum of silanol (*S*,*S*$_{Si}$)-1

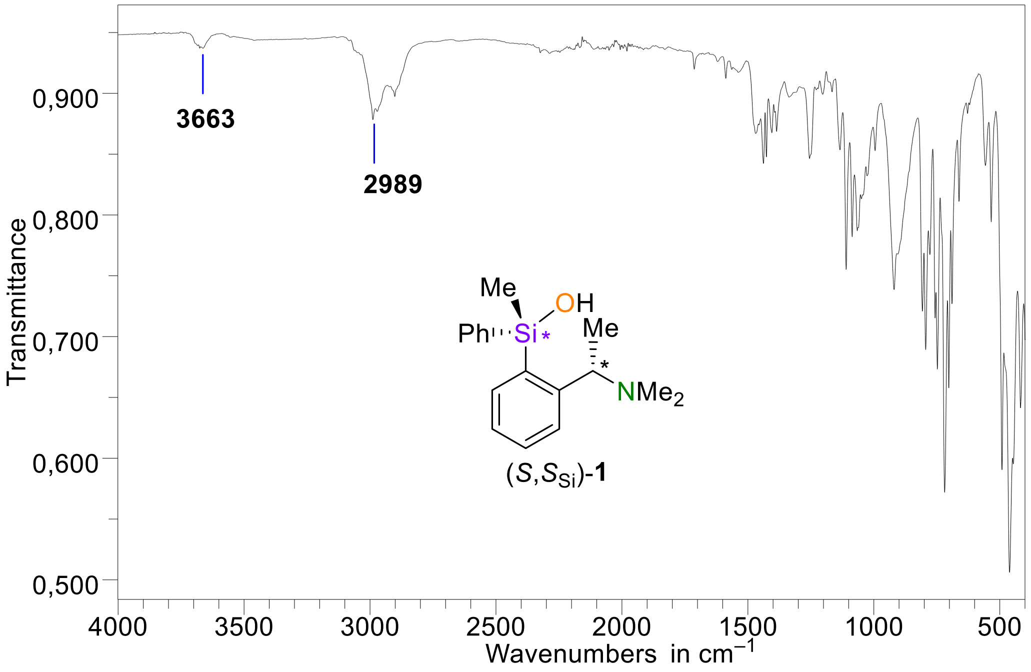

8.2. IR spectrum of zinc complex (S,S,S_{Si},S_{Si})-6

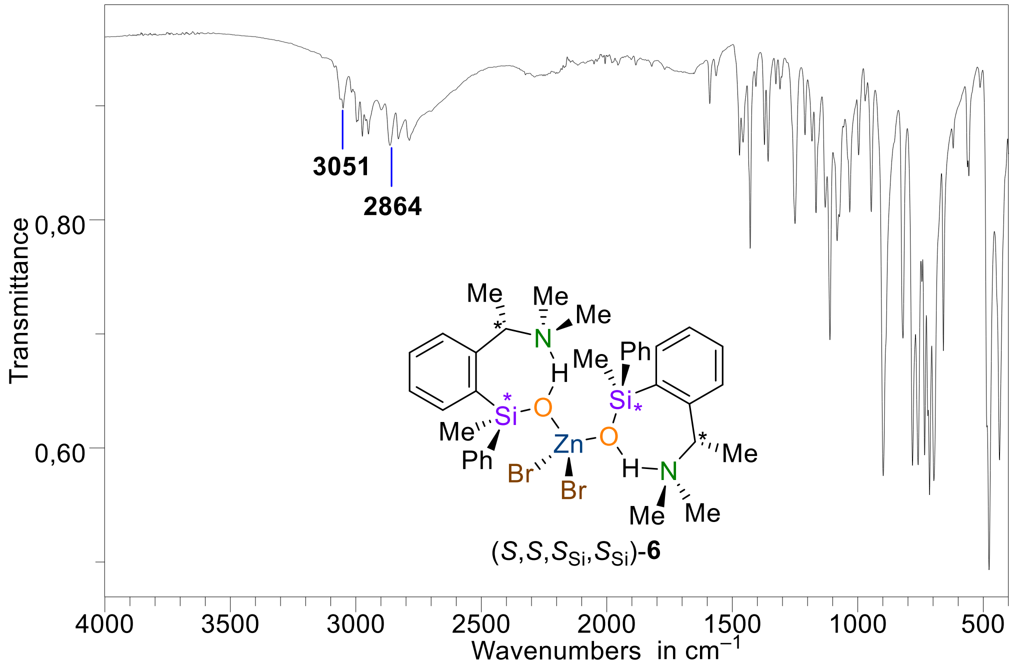

9. References

[1] Bruker, *APEX3*, Bruker AXS Inc., Madison, Wisconsin, USA, **2018**.

[2] O. V. Dolomanov, L. J. Bourhis, R. J. Gildea, J. A. K. Howard, H. Puschmann, J., *Appl. Crystallogr.* **2009**, *42*, 339–341.

[3] G. M. Sheldrick, *Acta crystallographica. Section A, Foundations and advances* **2015**, *71*, 3–8.

[4] G. M. Sheldrick, *Acta crystallographica. Section C, Structural chemistry* **2015**, *71*, 3–8.

[5] T. Weigel, C. Funke, M. Zschornak, T. Behm, H. Stöcker, T. Leisegang, D.C. Meyer, *J. Appl. Cryst.* **2020**, *53*, 614–622.

[6] I. N. Shishkina, V. M. Dem'yanovich, A. A. Kuznetsova, K. A. Potekhin, N. S. Zefirov, *Dokl Chem* **2008**, *423*, 279–282.
